# Highly Efficient Nitrogen Removal by *Stutzerimonas stutzeri* Strain MJ20: Metabolic Pathways and Potential for Biofloc Systems and Low C/N Ratio Aquaculture Wastewater

**DOI:** 10.3390/microorganisms14050975

**Published:** 2026-04-26

**Authors:** Miao Xie, Yongkui Liu, Chongqing Wen, Jiayi Zhong, Huanying Pang, Jia Cai, Yishan Lu, Jichang Jian, Yu Huang

**Affiliations:** 1 Guangdong Provincial Key Laboratory of Aquatic Animal Disease Control and Healthy Culture, College of Fishery, Guangdong Ocean University, Zhanjiang 524088, China; wonderful-520@126.com (M.X.); chongqingwen@163.com (C.W.); 18316693893@163.com (J.Z.); panghy@gdou.edu.cn (H.P.); caijia@gdou.edu.cn (J.C.); luys@gdou.edu.cn (Y.L.); 2 Key Laboratory of Diseases Controlling for Aquatic Economic Animals of Guangdong Higher Education Institutions, Zhanjiang 524088, China; 3 Southern Marine Science and Engineering Guangdong Laboratory, Zhanjiang 524025, China; liuyongkui@zjblab.com; 4 Guangdong Provincial Engineering Research Center for Aquatic Animal Health Assessment, Shenzhen 518000, China

**Keywords:** HNAD bacteria, *Stutzerimonas stutzeri*, nitrogen removal, biofloc technology

## Abstract

Although numerous studies have focused on the potential application of heterotrophic nitrification–aerobic denitrification (HNAD) bacteria in wastewater treatment, research exploring their potential in aquaculture biofloc systems remains limited. In this study, a promising HNAD strain, identified as *Stutzerimonas stutzeri* MJ20, was isolated from mature biofloc. This strain efficiently utilized low-cost carbon sources (e.g., glucose) and small-molecule carbon sources (e.g., sodium acetate and sodium succinate). Under conditions with glucose as the carbon source, a carbon-to-nitrogen (C/N) ratio of 15, pH 6–9, temperature 25–35 °C, salinity 0–35‰, and shaker speed of 0–150 rpm, it achieved removal rates of 95–100% for NH_4_^+^-N, NO_2_^−^-N, and NO_3_^−^-N at initial concentrations of 100 mg/L each. Even at higher concentrations (up to 200 mg/L NH_4_^+^-N and 500 mg/L for both NO_2_^−^-N and NO_3_^−^-N), removal rates exceeded 99%. Under mixed nitrogen sources, strain MJ20 demonstrated efficient nitrogen removal, preferentially utilizing NH_4_^+^-N, with only minimal and transient accumulation of nitrite and nitrate. Genomic analysis revealed that MJ20 carries key denitrification genes, including *napA*, *nirS*, *norB* and *nosZ*, and possesses complete pathways for nitrate reduction to nitrogen gas and ammonia assimilation, although typical autotrophic nitrification genes were not detected. Combined genomic data and autotrophic culture experiments indicated that, in addition to utilizing various organic carbon sources, the strain also exhibited certain autotrophic growth capabilities. Furthermore, MJ20 showed strong flocculation ability (flocculation rate > 96% within 16 h), sensitivity to multiple common antibiotics, and no toxicity to zebrafish, demonstrating favorable biosafety. In simulated seawater aquaculture wastewater with a C/N ratio of 5, it achieved a total nitrogen removal rate exceeding 94% within 72 h. These results indicate that strain MJ20 possesses comprehensive advantages, including efficient nitrogen removal, broad carbon source adaptability, strong environmental resilience, minimal accumulation of intermediate nitrogen products, excellent flocculation ability, and high biosafety. These traits highlight its potential for application in biofloc systems and in treating aquaculture tail water with a low C/N ratio. This study provides theoretical insights and practical guidance for screening HNAD bacteria suitable for biofloc systems.

## 1. Introduction

Biofloc Technology (BFT) serves as an effective method for removing toxic nitrogenous compounds in intensive aquaculture systems. This removal process is not accomplished by a single microorganism but relies on a consortium involved in the nitrogen cycle, including autotrophic nitrifying bacteria, heterotrophic bacteria, anaerobic denitrifying bacteria, and other microbial communities [1]. Previous studies have confirmed that heterotrophic nitrification–aerobic denitrification (HNAD) bacteria, capable of performing both nitrification and denitrification, play a crucial role in nitrogen recycling within BFT-based aquaculture systems [2,3].

Autotrophic nitrifying bacteria, the core agents of traditional nitrification, exhibit high substrate specificity and conversion efficiency. They do not require organic carbon but need supplemental alkalinity to maintain system stability. However, their growth is slow, with long generation times, and they are highly sensitive to environmental factors such as pH, temperature, dissolved oxygen, and heavy metal ions [4]. Additionally, they often require attachment to bio-carriers for efficient activity [5]. Consequently, in BFT systems, it takes a prolonged period for autotrophic nitrifiers to proliferate and become the dominant community. In contrast, HNAD bacteria are heterotrophic microorganisms that require organic carbon. They are characterized by rapid growth, high nitrogen conversion efficiency, and strong resistance to environmental stress. Unlike the two-step autotrophic nitrification performed separately by ammonia-oxidizing bacteria (AOB) and nitrite-oxidizing bacteria (NOB), HNAD bacteria can conduct both nitrification and denitrification within a single cell, thereby directly removing nitrogen from water. Compared to their autotrophic counterparts, HNAD bacteria demonstrate greater tolerance to fluctuations in dissolved oxygen, pH, temperature, and salinity, offering a broader application potential [6,7]. Some HNAD strains also possess flocculation ability [8]; they can secrete extracellular polymeric substances (EPS) to form larger, settleable flocs. This not only provides attachment sites for autotrophic nitrifiers but also creates oxygen-gradient microenvironments conducive for themselves and anaerobic denitrifiers, facilitating simultaneous nitrification and denitrification (SND). Therefore, HNAD bacteria are highly suitable as probiotics for biofloc formation. However, their metabolic pathways, particularly for carbon and nitrogen, are highly diverse [9]. Different strains vary significantly in their efficiency to utilize organic carbon sources, their abundance of nitrogen metabolism-related genes, and consequently, their nitrogen removal efficiency. Thus, careful screening of HNAD strains is essential for their effective application in BFT.

While sucrose, glucose, and starch are common, low-cost carbon sources used in BFT systems [10,11,12], many HNAD bacteria preferentially utilize simpler compounds like sodium acetate, succinate, or citrate, showing relatively lower efficiency with sucrose or starch [9]. Therefore, screening for HNAD strains capable of efficiently using low-cost carbon sources is a primary objective. An ideal HNAD candidate for BFT should also possess: (1) strong and complete heterotrophic ammonia assimilation and aerobic denitrification capabilities to rapidly remove NH_4_^+^-N, NO_2_^−^-N, and NO_3_^−^-N; (2) minimal nitrite accumulation during the process, as nitrite buildup is common during in situ biofloc cultivation [13]; (3) robustness to tolerate fluctuations in environmental factors like pH, temperature, salinity, and dissolved oxygen while maintaining efficient nitrogen removal; (4) strong flocculation activity to aid rapid biofloc formation; and (5) biosafety, being non-pathogenic and potentially beneficial to the cultured species.

Most prior research has focused on screening HNAD strains for industrial and agricultural wastewater treatment [6,14,15,16], with few reports targeting strains specifically for BFT application. To address this gap, this study aimed to isolate and characterize an HNAD strain that is not only efficient in nitrogen removal but also possesses strong flocculation capacity and good biosafety, with the goal of developing a potential microbial agent for seeding and maintaining BFT systems. Such a strain would also hold promise for treating aquaculture wastewater.

The primary scientific objective was to screen a HNAD bacterium capable of utilizing low-cost carbon sources while exhibiting high nitrogen removal efficiency, strong flocculation ability and biosafety, and to elucidate its metabolic mechanisms and potential application in BFT and wastewater treatment. To achieve this goal, we pursued four specific activities: (1) investigating its nitrogen removal performance under different environmental factors (carbon source, carbon-to-nitrogen (C/N) ratio, pH, temperature, salinity, dissolved oxygen, initial nitrogen concentration); (2) assessing its denitrification efficiency and conducting a nitrogen mass balance under single and combined nitrogen substrates; (3) exploring its carbon and nitrogen metabolic pathways via whole-genome sequencing; and (4) evaluating its application potential in BFT through flocculation rate assays, antibiotic susceptibility testing, biosafety assessment, and aquaculture wastewater treatment experiments. The findings are expected to provide theoretical insights and a practical foundation for the development of biofloc technology and the treatment of aquaculture wastewater.

## 2. Materials and Methods

### 2.1. Media Preparation

The enrichment medium (EM) used for HNAD strains screening consisted of (g/L): sucrose (27.292), NaNO_2_ (1.0), (NH_4_)_2_SO_4_ (2.0), KNO_3_ (1.0), K_2_HPO_4_ (1.0), MgSO_4_·7H_2_O (0.5), NaHCO_3_ (1.5), and trace-element liquor (5 mL). The final pH was adjusted to 8.2–8.5. The medium was sterilized by autoclaving at 115 °C and 0.1 MPa for 30 min. After cooling, the trace element solution was aseptically added.

Bromothymol blue (BTB) denitrification medium used for HNAD strains screening consisted of (g/L): tryptone (10.0), yeast extract (5.0), sodium chloride (5.0), bromothymol blue (BTB) indicator (1.0 mL), KNO_3_ (1.0), agar powder (20.0), trace-element liquor (5 mL). The final pH was 7.0–7.2. The medium (excluding the BTB indicator and trace element solution) was sterilized by autoclaving at 121 °C and 0.1 MPa for 20 min. After cooling, the sterile BTB indicator and trace element solution were aseptically added.

The basal denitrification medium (BDM) used for HNAD strains screening consisted of (g/L): NaCl (0.3), K_2_HPO_4_ (1.0), MgSO_4_·7H_2_O (0.5), NaHCO_3_ (1.5), and trace-element liquor (5 mL). Sodium acetate was added as organic carbon source to achieve a carbon concentration of 1500 mg/L. (NH_4_)_2_SO_4_, NaNO_2_, or KNO_3_ were added respectively as inorganic nitrogen sources to achieve a nitrogen concentration of 100 mg/L before use. The final pH was 8.2–8.5. The basal medium was sterilized by autoclaving at 121 °C and 0.1 MPa for 20 min. After cooling, the sterile trace element solution was aseptically added.

Luria–Bertani (LB) medium consisted of (g/L): tryptone (10), yeast extract (5), sodium chloride (10), and agar powder (10). The medium was sterilized by autoclaving at 121 °C and 0.1 MPa for 20 min.

The trace-element liquor consisted of (g/L): ZnSO_4_·7H_2_O (3.916), CaCl_2_ (5.5), MnSO_4_·H_2_O (6.85), FeSO_4_·7H_2_O (9.12), CuSO_4_ (1.57), CoCl_2_·6H_2_O (1.61), (NH_4_)_6_Mo_7_O_24_·4H_2_O (1.1), EDTA-2Na (5.71) [14]. It was sterilized by filtration through a 0.22 μm cellulose acetate membrane filter (Jinteng, Tianjin, China) prior to use and added to the sterilized and cooled culture medium after filter sterilization.

All chemical reagents were of analytical grade and were purchased from Sinopharm Chemical Reagent Co., Ltd. (Shanghai, China), unless otherwise stated. Tryptone, yeast extract, and agar were obtained from Beijing Land Bridge Technology Co., Ltd. (Beijing, China).

### 2.2. Isolation of HNAD Strains

Mature bioflocs used for the screening of HNAD strains were collected from an indoor experimental shrimp aquaculture workshop at Guangdong Ocean University, located in Mazhang District, Zhanjiang City, Guangdong Province, China (GPS coordinates: 21.15° N, 110.31° E). Specifically, during the mature phase, a 250 mL water sample was collected from a depth of 20 cm at five locations (the four corners and the center of the tank). For each location, 50 mL was collected, and the five aliquots were then combined to form a single composite sample. Water samples were pre-filtered with qualitative filter paper to remove coarse solids, and the resulting filtrate was passed through a 0.22 µm polyvinylidene fluoride (PVDF) membrane (Jinteng, Tianjin, China); subsequently, the residues from both filtration steps were combined and inoculated into the sterile enrichment medium. To dissociate the flocs and release attached microorganisms, sterile glass beads were added to the medium. The culture was incubated in an orbital shaker (Model DZP-2F, Changzhou Zhengrong Instrument Co., Ltd., Changzhou, China) at 30 °C and 160 rpm. Unless otherwise stated, an orbital shaker was used for all subsequent incubation steps in this study. The concentrations of ammonium nitrogen (NH_4_^+^-N), nitrite (NO_2_^−^-N), and nitrate (NO_3_^−^-N) in the medium were monitored daily using colorimetric test kits (Fishdoctor, Yancheng, China). The enrichment process was sustained for 30 days. To monitor metabolic progression, colorimetric tests were performed every 3 days for the first 20 days, then every 2 days for the final 10 days of incubation. Throughout this period, whenever the colorimetric tests indicated the depletion of all three aforementioned nitrogen species (i.e., showed negative results), sucrose and the nitrogen sources were replenished to re-establish a C/N ratio of 15:1. Simultaneously, sodium bicarbonate was added to maintain the pH within the range of 8.2 to 8.5.

Following enrichment, 1 mL of the culture was serially diluted, spread onto BTB denitrification medium plates, and incubated at 30 °C for 72 h. Single colonies surrounded by a blue halo, indicative of denitrification and alkalinization, were selected as potential HNAD isolates. These colonies were purified by repeated streaking (five times) on LB agar plates. Subsequently, isolated colonies were inoculated into 1.5 mL centrifuge tubes containing a basal denitrification medium (BDM) and incubated for 48 h. The concentrations of NH_4_^+^-N, NO_2_^−^-N, and NO_3_^−^-N in the supernatants were determined using a UV-Vis spectrophotometer (Model UV-5300, Shanghai Yuanxi Instrument Co., Ltd., Shanghai, China) according to standard colorimetric methods [17]. Unless otherwise stated, this instrument was used for all subsequent spectrophotometric measurements in this study. Furthermore, the flocculation activity of the isolated strains was evaluated with the kaolin clay assay [18]. Strains exhibiting complete removal of 100 mg/L of each nitrogen species (NH_4_^+^-N, NO_2_^−^-N, and NO_3_^−^-N) within 24 h, coupled with significant flocculation activity, were shortlisted for further analysis. Through this screening process, a promising HNAD strain was isolated based on its superior nitrogen removal performance and flocculation activity. The pure culture of this isolated strain was preserved in 25% sterile glycerol at −80 °C for all subsequent experiments. Specifically, the storage medium consisted of an equal volume mixture of 50% sterile glycerol and bacterial suspension harvested from the logarithmic growth phase.

### 2.3. Identification of HNAD Strains

The morphology of the strain showing superior nitrogen removal performance was examined using Gram staining followed by light microscopy. Its phenotypic characteristics, particularly the carbon source utilization profile, were investigated using Biolog GEN III microplates (Biolog, Hayward, CA, USA) according to the manufacturer’s instructions. Genomic DNA was extracted from the isolated strain using a Bacterial Genomic DNA Extraction Kit (TIANGEN, Beijing, China). The nearly full-length 16S rDNA gene was amplified via polymerase chain reaction (PCR) with the universal bacterial primers 27F (5′-AGAGTTTGATCCTGGCTCAG-3′) and 1492R (5′-GGTTACCTTGTTACGACTT-3′). The PCR products were verified by 1% agarose gel electrophoresis and subsequently sent to Sangon Biotech Co., Ltd. (Shanghai, China) for Sanger sequencing. The obtained 16S rRNA sequence was submitted to the GenBank database and analyzed via the web-based version of BLAST (http://blast.ncbi.nlm.nih.gov/Blast.cgi, accessed on 26 January 2026). A phylogenetic tree was constructed using MEGA software (version 7.0) based on the aligned 16S rDNA sequence of the isolated strain and closely related reference sequences retrieved from the database, illustrating the phylogenetic kinship of the isolate.

### 2.4. Optimization of Factors Affecting Nitrogen Removal Performance

To determine the optimal culture conditions for the isolated strain, seven single-factor experiments was conducted using a BDM with different inorganic nitrogen sources. The effects of the following environmental factors on bacterial growth and nitrogen removal efficiency were investigated: carbon source (sucrose, glucose, starch, sodium acetate, sodium succinate, sodium citrate), C/N ratio (0, 2, 5, 10, 15, 20, 25, 30), temperature (15, 20, 25, 30, 35, 40, 45 °C), initial pH (5.0, 6.0, 7.0, 8.0, 9.0, 10.0), salinity (0, 5, 10, 15, 20, 25, 30, 35, 40, 45, 50‰), shaker speed (0, 50, 100, 150, 200 rpm), and initial nitrogen concentration (100, 200, 300, 400, 500, 600, 800, 1000 mg/L). To account for abiotic nitrogen loss or fluctuation, uninoculated sterile controls were prepared for each set of experiments. All treatments (including controls) were performed in triplicate (*n* = 3).

The seven environmental factors and their gradients were selected based on typical aquaculture conditions and the known characteristics of HNAD bacteria to ensure practical relevance. Specifically, temperature (15–45 °C), salinity (0–50‰), and pH (5.0–10.0) were selected to cover the wide-ranging fluctuations observed in various aquaculture systems, while dissolved oxygen levels (0–200 rpm) simulated the transition from anaerobic to aerobic conditions. And the specific carbon sources and C/N ratios (0–30) were selected to evaluate the strain’s capacity to efficiently utilize low-cost carbon substrates and its denitrification performance under various C/N conditions typical of biofloc systems and aquaculture wastewater. Additionally, the high initial nitrogen concentrations (100–1000 mg/L) were selected to rigorously assess the strain’s tolerance and efficiency in highly polluted wastewater.

Before inoculation, a pre-cultured suspension of the isolated strain (24-h culture) was centrifuged (Model JIDI-17R, Guangzhou Jidi Instrument Co., Ltd., Guangzhou, China) at 12,000× *g* for 5 min; unless otherwise stated, this instrument was used for all subsequent centrifugation in this study. The pellet was washed three times with sterile phosphate-buffered saline (PBS) and resuspended to an optical density at 600 nm (OD_600_) of 1.5, measured using a bacterial turbidimeter (Model WGZ-2XJ, Shanghai Xinrui Instrument & Meter Co., Ltd., Shanghai, China); unless otherwise stated, this instrument was used for all subsequent bacterial growth density measurements in this study. This cell suspension was inoculated at 0.1% (*v*/*v*) into 100 mL bottles containing 40 mL of BDM. The composition and initial pH of the BDM were adjusted according to the requirements of each specific single-factor test. The carbon source screening experiment was performed under baseline conditions of 30 °C, 150 rpm, and a C/N ratio of 15. All other single-factor experiments were subsequently conducted by varying one parameter at a time based on these baseline conditions and the optimal result from the preceding test. After 24 h of incubation under the specified conditions, bacterial growth (OD_600_) and the residual concentrations of ammonium, nitrite, and nitrate were measured. The efficient utilization of the nitrogen substrate was evaluated by calculating the nitrogen removal efficiency. The calculation was performed using Equation (1):

Nitrogen Removal Efficiency (%) = (1 − *C_t_*/*C*_0_) × 100% (1)

where *C_t_* is the residual nitrogen concentration in the experimental group inoculated with the isolated strain, *C*_0_ is the nitrogen concentration in the control group.

### 2.5. Nitrogen Removal Performance Assessment with Various Nitrogen Source

Following the determination of optimal conditions via single-factor experiments, the nitrogen removal capability of the isolated strain was further evaluated under these optimized culture parameters (glucose served as the sole carbon source, C/N = 15, pH 8, 30 °C, salinity 5‰, 150 rpm). Experiments were designed to test the strain’s performance with different nitrogen substrates, including three single nitrogen sources (ammonium sulfate, sodium nitrite, and potassium nitrate) and four combined nitrogen sources: (1) ammonium sulfate + sodium nitrite, (2) ammonium sulfate + potassium nitrate, (3) sodium nitrite + potassium nitrate, and (4) a mixture of all three. To account for abiotic nitrogen loss or fluctuation, uninoculated sterile controls were prepared for each set of experiments. All treatments (including controls) were performed in triplicate (*n* = 3).

The initial concentrations for the single nitrogen source groups were set at approximately 118.02 mg/L for NH_4_^+^-N, 113.91 mg/L for NO_2_^−^-N, and 101.17 mg/L for NO_3_^−^-N. For the two-component mixed nitrogen sources, the total nitrogen concentration was set to half the sum of the concentrations of the two corresponding single sources. For the three-component mixture, the total nitrogen concentration was one-third of the sum of the three single source concentrations. This design maintained a comparable total nitrogen load across different substrate conditions for performance comparison. Bacterial growth (OD_600_) and the residual concentrations of all relevant nitrogen species (NH_4_^+^-N, NO_2_^−^-N, NO_3_^−^-N) were monitored and measured at 4-h intervals over a 24-h incubation period. The experimental duration was divided into six distinct intervals: 0–4 h, 4–8 h, 8–12 h, 12–16 h, 16–20 h, and 20–24 h. The specific nitrogen removal rate (R, mg/L/h) for each interval was calculated based on the linear regression of nitrogen concentration depletion over time. The maximum degradation rate was identified by comparing the calculated R values across all six intervals. The calculation was conducted using Equation (2):

*R* = −(*C*_2_ − *C*_1_)/(*t*_2_ − *t*_1_) (2)

where *C*_1_ and *C*_2_ represent the nitrogen concentrations (mg/L) at the beginning and end of each time interval, and *t*_1_ and *t*_2_ represent the corresponding sampling times (h).

### 2.6. Calculation of Nitrogen Balance

A nitrogen mass balance was conducted to trace the fate of nitrogen within the reaction system. The total nitrogen was partitioned into three fractions: (1) biomass nitrogen (N_Bio_), representing nitrogen assimilated into bacterial cells; (2) dissolved total nitrogen (DTN) in the aqueous phase, comprising NH_4_^+^-N, NO_2_^−^-N, and NO_3_^−^-N; and (3) gaseous nitrogen products (N_g_), generated via denitrification. Bacterial cultures from the nitrogen source utilization experiment (Section 2.5) were collected at the initial (0 h) and final (24 h) time points. The same uninoculated sterile controls described in Section 2.5 were utilized to correct for abiotic nitrogen changes. Samples were filtered through a 0.22 µm cellulose acetate membrane (Jinteng, Tianjin, China) to determine the concentrations of NH_4_^+^-N, NO_2_^−^-N, NO_3_^−^-N. Biomass nitrogen (N_Bio_) was determined based on the mass balance difference. Specifically, the nitrogen content associated with biomass at 0 h (N_Bio0_) and 24 h (N_Bio1_) was calculated by subtracting the total nitrogen concentration in the supernatant (after centrifugation at 4000× *g* for 15 min at 4 °C) from the total nitrogen concentration of the whole culture (before centrifugation) [19]. As no gas collection apparatus was used, the amount of gaseous nitrogen produced was estimated indirectly by the difference in the total nitrogen mass balance. The relevant parameters and calculation were performed using Equations (3)–(8):

DTN = ρ(NH_4_^+^-N) + ρ(NO_2_^−^-N) + ρ(NO_3_^−^-N) (3)

TN_i_ = DTN_i_ + N_Bio0_ (4)

TN_f_ = DTN_f_ + N_Bio1_ (5)

Ng = TN_i_ − TN_f_ (6)

Nitrogen assimilation efficiency (%) = [(N_Bio1_ − N_Bio0_)/DTN_i_] × 100% (7)

Total nitrogen removal efficiency (%) = [(N_g_ + (N_Bio1_ − N_Bio0_))/TN_i_] × 100% (8)

where ρ(NH_4_^+^-N), ρ(NO_2_^−^-N), and ρ(NO_3_^−^-N) are the concentrations (mg/L) of respective nitrogen species in the water. DTN_i_ and DTN_f_ are the initial and final dissolved total nitrogen concentrations (mg/L). TN_i_ and TN_f_ are the initial and final total nitrogen concentrations (mg/L).

### 2.7. Complete Genome Analysis of the Isolated Strain

The isolated strain was cultured in LB broth at 30 °C with shaking at 150 rpm until the logarithmic growth phase was reached. The cells were harvested by centrifugation at 5000× *g* for 5 min at 4 °C. The pellet was collected, immediately flash-frozen in liquid nitrogen, and stored at −80 °C. Frozen cell pellets were shipped on dry ice to BGI Genomics Co., Ltd. (Shenzhen, China) for whole-genome sequencing. Following sequencing, the raw reads were assembled de novo to generate a complete genome. Protein-coding genes were predicted and functionally annotated against several databases, including the Kyoto Encyclopedia of Genes and Genomes (KEGG), Non-Redundant Protein Database (NR), Gene Ontology (GO), Clusters of Orthologous Groups of proteins (COG), and Swiss-Prot. Based on the annotation results, the genomic basis underlying the nitrogen and carbon metabolic pathways of the isolated strain was investigated.

### 2.8. Flocculation Ability Analysis of the Isolated Strain

The isolated strain in the logarithmic growth phase was inoculated at 0.1% (*v*/*v*) into 200 mL of a basal denitrification medium for HNAD bacteria. Glucose served as the sole carbon source. Ammonium sulfate, sodium nitrite, and potassium nitrate were used as nitrogen sources, with the concentration of each nitrogen species set at 100 mg/L. The C/N ratio was adjusted to 15:1, and the initial pH was maintained at 8.0. The culture was incubated at 30 °C with shaking at 150 rpm. Samples were collected every 4 h to measure the flocculation rate. All assays were performed with three independent biological replicates.

The flocculation activity was quantified using the kaolin clay assay [18]. Briefly, 2 mL of the bacterial culture was transferred to a 150 mL conical flask. Then, 5 mL of a 1% (*w*/*v*) calcium chloride (CaCl_2_) solution and 46 mL of a 0.4% (*w*/*v*) kaolin suspension were added sequentially. The mixture was brought to a final volume of 100 mL with deionized water. The contents were rapidly stirred at 220 rpm on a magnetic stirrer for 1 min, followed by slow stirring at 100 rpm for 3 min. After allowing the mixture to settle for 10 min, a sample of the supernatant was carefully taken from 1 cm below the liquid surface. The absorbance of this supernatant (A) was measured at 550 nm. A control absorbance (B) was obtained by repeating the entire procedure using 2 mL of sterile, uninoculated LB medium instead of the bacterial culture. The flocculation rate was calculated using Equation (9):

Flocculation rate (%) = [(B − A)/B] × 100%(9)

### 2.9. Biosafety Analysis of the Isolated Strain

#### 2.9.1. Hemolysis Assay Determination

A single colony of the isolated strain was isolated from an LB nutrient agar plate and streaked onto a blood agar plate (BKMAMLAB, Changde, China). The plate was inverted and incubated at 37 °C for 48 h. Following incubation, hemolysis was observed by examining the plate against a light source from the reverse side. *Vibrio parahaemolyticus* was used as a positive control.

#### 2.9.2. Antimicrobial Susceptibility Evaluation

Susceptibility was determined using the standard disk diffusion method. Eighteen antibiotics (listed in the Results section) were selected based on those commonly used in aquaculture and clinical settings, as well as recommendations by the Clinical and Laboratory Standards Institute (CLSI). *Escherichia coli* ATCC 25922 was used as the quality control strain. The interpretation of susceptibility was performed according to the CLSI (35th ed., 2025) guidelines [20]. The results were categorized as Sensitive (S), Intermediate (I/M), and Resistant (R).

Briefly, a logarithmic-phase culture of the isolated strain was adjusted to an OD_600_ of 0.5. Then, 100 µL of the bacterial suspension was spread evenly onto the surface of an LB nutrient agar plate. After allowing the agar surface to dry for 5 min, antibiotic disks (Hangwei, Hangzhou, China) were placed on the plate in a triangular arrangement and gently pressed with sterile forceps to ensure full contact. Plates were incubated inverted at 37 °C for 24 h. The diameters of the inhibition zones were measured with a vernier caliper, and susceptibility was interpreted based on the zone size.

#### 2.9.3. Safety Evaluation in an Aquatic Animal Model

Healthy zebrafish (*Danio rerio*; 3–4 cm body length) were acclimated for one week before the experiment. Fish were randomly allocated into four groups and maintained in 10 L plastic tanks, including three experimental groups and one control group (10 fish per group). Room temperature was maintained at 28 °C using air conditioning. An overnight culture of isolated strain was added to the tank water to achieve a final concentration of 1 × 10^6^ CFU/mL. Fish were fed three times daily. The tank water was completely replaced every 24 h, and the bacterial suspension was replenished to restore the initial concentration. A control group received identical handling except for the addition of bacteria. Health status and mortality were recorded daily over a 10-day observation period.

### 2.10. Nitrogen Removal Assay Using Simulated Mariculture Wastewater

Simulated mariculture wastewater was formulated based on the characteristics of shrimp culture effluent as described by Fontenot [21] with some modifications. Its specific composition is detailed in Table 1. For the experimental groups, 500 mL of the simulated wastewater in 1 L Erlenmeyer flasks was inoculated with 1% (*v*/*v*) of an overnight culture of the isolated strain; control groups received no bacterial inoculum. Glucose was supplied as the sole carbon source to obtain a C/N ratio of 5:1. The flasks were incubated at 30 °C with shaking at 150 rpm for 72 h. Concentrations of NH_4_^+^-N, NO_2_^−^-N, NO_3_^−^-N, and TN were measured after being filtered through a 0.22 µm cellulose acetate membrane (Jinteng, Tianjin, China) at 8-h intervals throughout the incubation period. All assays were performed with three independent biological replicates.

### 2.11. Analytical Methods

Bacterial growth was monitored by measuring the optical density at 600 nm (OD_600_). The concentrations of NH_4_^+^-N, NO_2_^−^-N, NO_3_^−^-N, and total nitrogen (TN) were determined according to standard methods [17]. Specifically, NH_4_^+^-N was analyzed by Nessler’s reagent spectrophotometry, NO_2_^−^-N by the N-(1-naphthyl)-ethylenediamine spectrophotometric method, NO_3_^−^-N by ultraviolet (UV) spectrophotometry, and TN by the alkaline potassium persulfate digestion UV spectrophotometric method. Digestion was performed in a vertical autoclave (Model LDZX-50KBS, Shanghai Shen’an Medical Instrument Factory, Shanghai, China).

### 2.12. Statistical Analysis

Data are presented as the mean ± standard deviation. One-way analysis of variance (ANOVA) followed by Tukey’s post hoc test was performed to assess the statistical significance of the effects of environmental factors on the nitrogen-removal capacity of the isolated strain under oxic conditions. A *p*-value < 0.05 was considered statistically significant. Statistical analysis was conducted using Microsoft Excel 2017 (Microsoft Corporation, Redmond, WA, USA). Scatter plots and bar charts were generated using GraphPad Prism 10.4 (GraphPad Software, San Diego, CA, USA). The metabolic pathway diagram was created with Adobe Illustrator 2025 (Adobe Inc., San Jose, CA, USA).

## 3. Results and Discussion

### 3.1. Isolation and Identification of Strain MJ20

**Figure 1:**
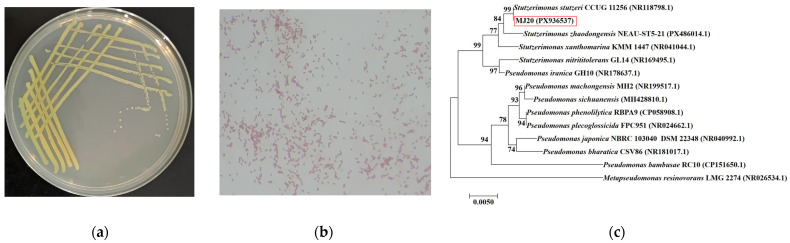
(**a**) Colony morphology image of strain MJ20; (**b**) Gram staining image of MJ20 (10 × 100); (**c**) Phylogenetic tree of strain MJ20 and its closely related model strains.

Eleven HNAD bacterial strains capable of nitrogen degradation were isolated from biofloc samples collected in a shrimp aquaculture workshop. Among them, strain MJ20, which exhibited high nitrogen degradation capability and flocculation activity, was selected as the target strain for further study. As depicted in Figure 1a, colonies of strain MJ20 cultured at 37 °C for 24 h appeared pale yellow, circular with entire margins, moist, and exhibited a smooth surface. The cells were Gram-negative short rods (Figure 1b). Partial 16S rDNA gene sequences of MJ20 were analyzed using the web-based version of BLAST tool in the NCBI database, resulting in the assignment of GenBank accession number PX936537. The BLAST results indicated that the sequences shared 99% identity with *Stutzerimonas stutzeri* CCUG 11256, placing the isolate within the genus *Stutzerimonas*. Phylogenetic analysis conducted with MEGA7.0 (Figure 1c) further confirmed the classification of strain MJ20 as *Stutzerimonas stutzeri*. Consequently, the strain was designated *Stutzerimonas stutzeri* MJ20. Notably, many HNAD bacteria applied in nitrogen contaminated wastewater belong to the genus *Pseudomonas* (now reclassified in part as *Stutzerimonas*), such as *P. stutzeri* XL-2 [22], *P. stutzeri* ATCC 17588 [23], *P. stutzeri* JCM20778 [24], *P. stutzeri* Y23 [25], and *Pseudomonassp.* Y15 [26].

Strain MJ20 was isolated from mature bioflocs in a shrimp aquaculture facility, and its source environment provides direct ecological context for its metabolic traits. Biofloc systems are characterized by high nitrogen loading, fluctuating dissolved oxygen levels, and intense microbial competition, which impose strong selective pressure. In this environment, MJ20 likely evolved efficient HNAD capabilities, enabling it to remove ammonia and nitrite effectively under high organic loads, thereby gaining a competitive edge. Moreover, its prolonged residence within the floc matrix may have endowed it with robust biofilm-forming and attachment potential, further supporting its practical applicability in biofloc-based water treatment systems. Thus, the isolation of MJ20 from bioflocs not only explains the origin of its exceptional nitrogen-metabolizing functions, but also suggests that it may exhibit enhanced adaptability and functional stability when reintroduced into similar engineered systems.

### 3.2. Physiological and Biochemical Characterization of Strain MJ20

**Figure 2:**
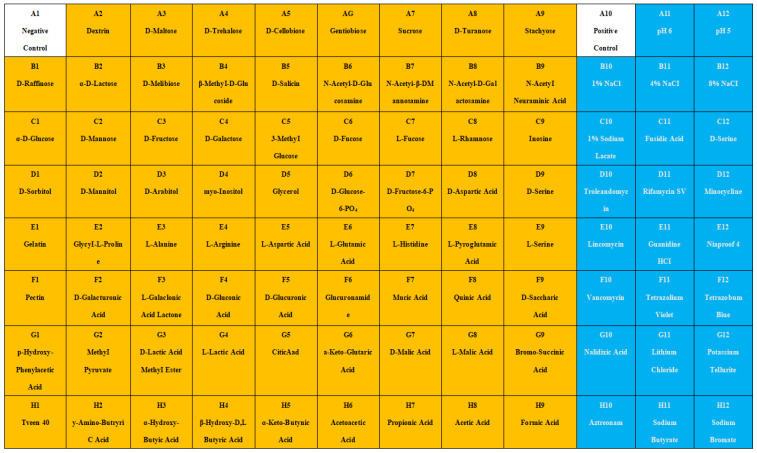
Testing layout map of Biolog Gen III Microplate. Note: Cells highlighted in orange are 71 carbon sources, and cells highlighted in blue are 23 chemical sensitivity tests. The white cells refer to the negative control (A1) and positive control (A10) wells.

**Figure 3:**
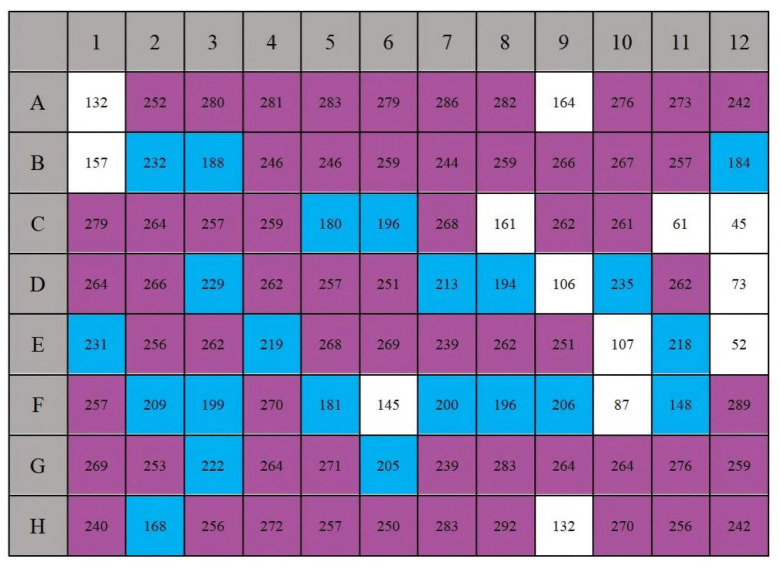
Colorimetric result of MJ20 strain on Biolog Gen III Microplate. Note: The purple boxes represent positive values; the blue boxes represent intermediate values; the white boxes represent negative values.

The physiological and biochemical profile of strain MJ20 was determined using the Biolog Gen III MicroPlate system, encompassing 94 phenotypic tests (71 carbon source utilization assays and 23 chemical sensitivity assays) (Figure 2). The identification results are presented in Figure 3. Phenotypic analysis revealed that strain MJ20 could utilize a wide spectrum of 65 carbon sources. This extensive metabolic repertoire is superior to that of many previously reported HNAD strains, such as *Rhodococcus* sp. [27], *Pseudomonas* sp. [24] and *Paracoccus* sp. [28], indicating a higher degree of versatility. These included common substrates such as dextrin, maltose, sucrose, glucose, fructose, and glycerol, as well as key tricarboxylic acid (TCA) cycle intermediates (e.g., citrate, α-ketoglutarate, and malate). This trait is highly advantageous for BFT, as MJ20 utilizes cost-effective sugars like glucose and sucrose, contrasting with *Pseudomonas stutzeri* UFV5′s preference for pricier organic acids like citrate and succinate [29]. Chemical sensitivity tests indicated that strain MJ20 was capable of growth in NaCl concentrations ranging from 1% to 8% and tolerated pH levels between 5 and 6, surpassing the adaptability of the strain *Pseudomonas aeruginosa* SH3 [29], which thrives within a narrower salinity range of 1.5% to 3.5% and a neutral-to-alkaline range pH 7–9. This broad tolerance makes it suitable for both low-salinity and marine shrimp farming, thereby enhancing its ecological competitiveness within complex aquatic microbial communities.

### 3.3. Effects of Environmental Factors on Nitrogen Removal of the Strain MJ20

#### 3.3.1. Carbon Source

**Figure 4:**
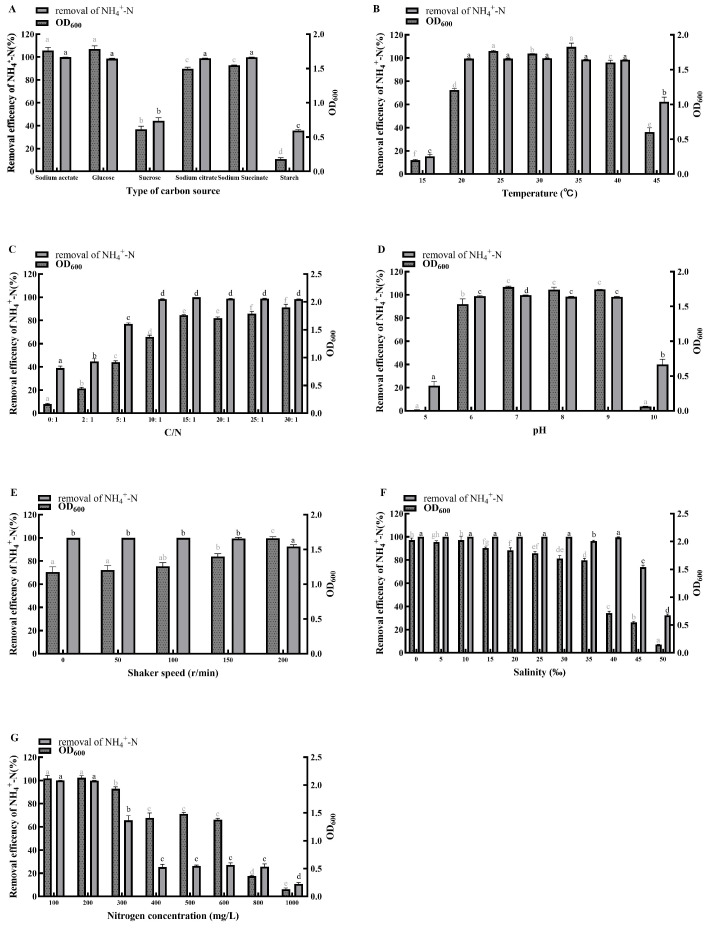
Effects of environmental factors on NH_4_^+^-N removal of the strain MJ20. (**A**) Types of carbon source (sodium acetate, glucose, sucrose, sodium citrate, sodium succinate, starch); (**B**) Temperature (15, 20, 25, 30, 35, 40, 45 °C); (**C**) C/N (0, 2, 5, 10, 15, 20, 25, 30); (**D**) pH (5.0, 6.0, 7.0, 8.0, 9.0, 10.0); (**E**) shaker speed (0, 50, 100, 150, 200 rpm); (**F**) Salinity (0, 5, 10, 15, 20, 25, 30, 35, 40, 45, 50‰); (**G**) Nitrogen concentration (100, 200, 300, 400, 500, 600, 800, 1000 mg/L). Treatments labeled with different letters are significantly different (*p* < 0.05).

**Figure 5:**
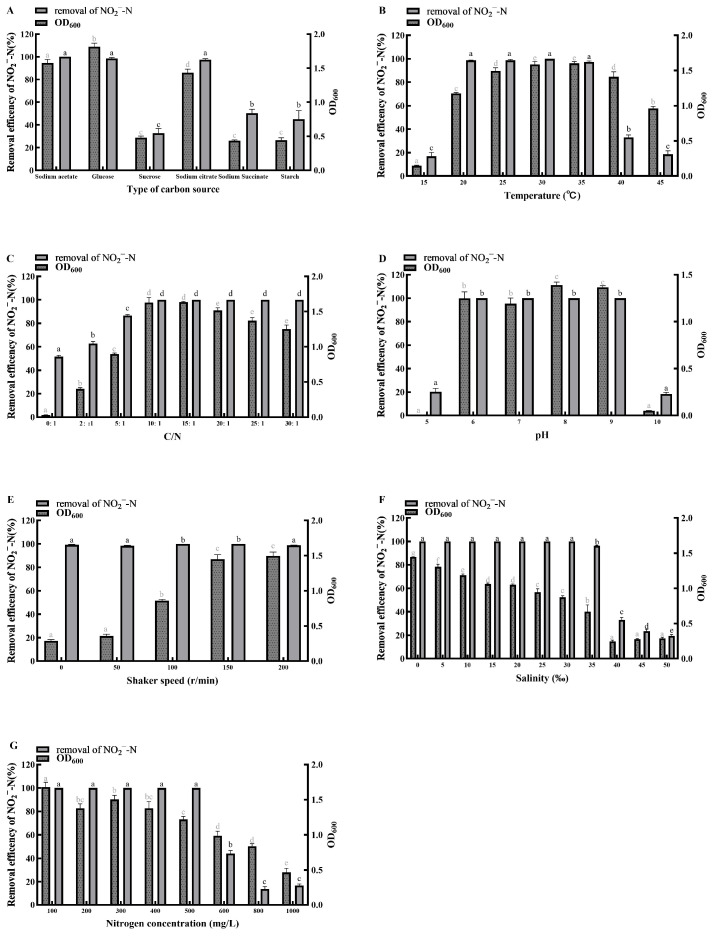
Effects of environmental factors on NO_2_^−^-N removal of the strain MJ20. (**A**) Types of carbon source (sodium acetate, glucose, sucrose, sodium citrate, sodium succinate, starch); (**B**) Temperature (15, 20, 25, 30, 35, 40, 45 °C); (**C**) C/N (0, 2, 5, 10, 15, 20, 25, 30); (**D**) pH (5.0, 6.0, 7.0, 8.0, 9.0, 10.0); (**E**) shaker speed (0, 50, 100, 150, 200 rpm); (**F**) Salinity (0, 5, 10, 15, 20, 25, 30, 35, 40, 45, 50‰); (**G**) Nitrogen concentration (100, 200, 300, 400, 500, 600, 800, 1000 mg/L). Treatments labeled with different letters are significantly different (*p* < 0.05).

**Figure 6:**
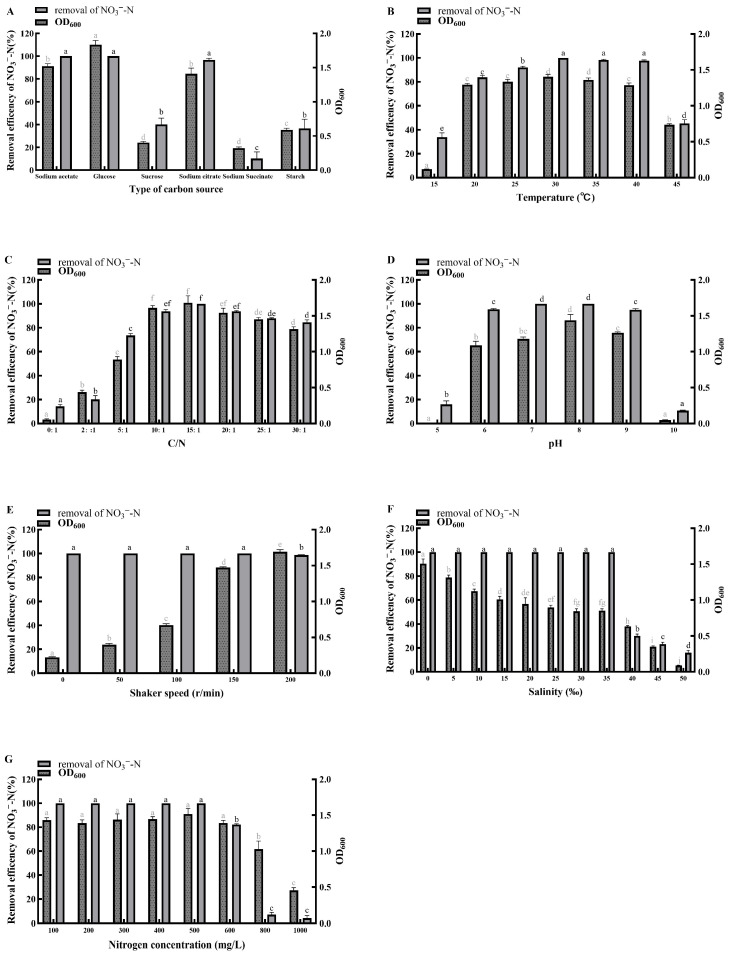
Effects of environmental factors on NO_3_^−^-N removal of the strain MJ20. (**A**) Types of carbon source (sodium acetate, glucose, sucrose, sodium citrate, sodium succinate, starch); (**B**) Temperature (15, 20, 25, 30, 35, 40, 45 °C); (**C**) C/N (0, 2, 5, 10, 15, 20, 25, 30); (**D**) pH (5.0, 6.0, 7.0, 8.0, 9.0, 10.0); (**E**) shaker speed (0, 50, 100, 150, 200 rpm); (**F**) Salinity (0, 5, 10, 15, 20, 25, 30, 35, 40, 45, 50‰); (**G**) Nitrogen concentration (100, 200, 300, 400, 500, 600, 800, 1000 mg/L). Treatments labeled with different letters are significantly different (*p* < 0.05).

When sodium acetate, glucose, or sodium citrate was supplied as the sole carbon source, the removal rates of NH_4_^+^-N (Figure 4A), NO_2_^−^-N (Figure 5A), and NO_3_^−^-N (Figure 6A) reached 95–100% within 24 h, accompanied by cell growth (OD_600_) of 1.4–1.8. The glucose group showed the highest growth, with OD_600_ exceeding 1.8. In contrast, when sucrose or starch served as the sole carbon source, the removal of all three nitrogen forms was below 50% within 24 h, and OD_600_ remained under 1.0. Nevertheless, the sucrose group achieved complete removal (100%) of all three nitrogen forms by 48 h. When sodium succinate was used as the sole carbon source, NH_4_^+^-N removal exceeded 99% within 24 h, while the removal rates of NO_2_^−^-N and NO_3_^−^-N were only about 50% and 10%, respectively. Therefore, for efficient and rapid nitrogen removal by strain MJ20, suitable carbon sources include sodium acetate, glucose, and sodium citrate, with glucose being the optimum choice.

These findings confirm previous reports that HNAD bacteria preferentially utilize small-molecule organic carbon sources such as sodium acetate, sodium citrate, and sodium succinate [30,31]. These compounds are intermediates of the TCA cycle and can be readily assimilated by strain MJ20, entering the central metabolism for rapid energy generation. Notably, strain MJ20 not only utilizes typical small-molecule carbon sources like sodium acetate but also efficiently utilizes low-cost substrates such as glucose, which may help reduce the operational cost of biofloc cultivation in practical applications.

#### 3.3.2. Temperature

Strain MJ20 was capable of growing within a temperature range of 15 °C to 45 °C. It exhibited efficient removal of NH_4_^+^-N (Figure 4B) and NO_3_^−^-N (Figure 6B), along with rapid cell growth, at temperatures between 20 °C and 40 °C. In contrast, at 15 °C and 45 °C, the removal rates of NH_4_^+^-N (Figure 5B) were only 15.39% and 62.29%, respectively, while those of NO_3_^−^-N were 33.78% and 45.31%. Meanwhile, a high NO_2_^−^-N removal efficiency was maintained within the range of 97.30–100% at temperatures between 20 °C and 35 °C. Temperatures above 35 °C led to markedly reduced NO_2_^−^-N removal and slower cell growth, which can be attributed to the increased bio-toxicity of nitrite under elevated temperature conditions [32].

These results are consistent with previous reports that most HNAD bacteria are mesophiles, with an optimal growth temperature range of 25–37 °C [6,33]. The present study further specifies that strain MJ20 retains robust nitrogen removal performance within a relatively wide window of 20–40 °C. Notably, this optimal temperature range aligns well with the suitable water temperatures (typically 20–30 °C) for the cultivation of many major aquaculture species, such as tilapia [34], carp [35], and shrimp [36]. This congruence suggests that strain MJ20 possesses inherent physiological compatibility for potential application in in situ bioremediation or biofloc technology within aquaculture systems, without requiring extensive temperature adjustment.

#### 3.3.3. C/N Ratio

Strain MJ20 was capable of growing across a C/N ratio range of 0 to 30. It demonstrated robust nitrogen removal performance and rapid cell growth as the C/N ratio increased from 0 to 15. However, a further increase in the C/N ratio beyond 15 led to a slight decline in NH_4_^+^-N removal, a significant decrease in NO_2_^−^-N removal, and a marked reduction in both NO_3_^−^-N removal and cell growth. Notably, at a C/N ratio of 5, strain MJ20 achieved over 70% removal efficiency for NH_4_^+^-N (Figure 4C), NO_2_^−^-N (Figure 5C), and NO_3_^−^-N (Figure 6C). Even under a C/N ratio of 0, it maintained a NO_2_^−^-N removal efficiency exceeding 50%. This indicates a strong adaptability to low C/N conditions and suggests potential autotrophic or mixotrophic capabilities, which could facilitate its application in autotrophic biofloc cultivation and in treating wastewater characterized by a low C/N ratio. These observations are generally consistent with previous reports that a C/N ratio above 7 is beneficial for the growth and nitrogen removal of most HNAD bacteria, with an optimal range often between 8 and 15 [9]. The performance decline observed at excessively high C/N ratios (e.g., 30) may be attributed to factors such as microbial metabolic imbalance or, as suggested by prior studies, potential cell lysis [33].

#### 3.3.4. pH

Strain MJ20 exhibited high nitrogen removal efficiency and rapid cell growth within the pH range of 6.0 to 9.0, achieving removal rates of 95–100% for NH_4_^+^-N (Figure 4D), NO_2_^−^-N (Figure 5D), and NO_3_^−^-N (Figure 6D). In contrast, at pH 5.0 and 10.0, both nitrogen removal and cell growth decreased sharply, with bacterial growth nearly ceasing. Although HNAD bacteria can be found in diverse acidic, neutral, and alkaline environments, most exhibit a preference for weakly alkaline conditions, with an optimal pH range for nitrogen removal typically between 7.0 and 8.0 [9]. The results indicate that strain MJ20 is capable of surviving and performing efficiently across a broad spectrum from weakly acidic to weakly alkaline conditions. Therefore, the suitable pH range for achieving efficient nitrogen removal with strain MJ20 is 6.0–9.0. This range aligns well with the optimal pH conditions (commonly pH 6.5–8.5) required for the health and growth of most cultured aquatic species [37]. This shared optimal pH window is particularly advantageous, as it simultaneously supports high nitrogen removal activity and safeguards the physiological health and stress resistance of farmed animals, thereby promoting the overall stability of the aquaculture system.

#### 3.3.5. Shaker Speed

Dissolved oxygen (DO) concentration is a critical parameter for nitrogen removal in the denitrification process and is positively correlated with shaker speed [38]. Strain MJ20 was capable of growing under shaker speeds ranging from 0 to 200 rpm, with its growth yield increasing as the shaker speed rose. It achieved removal rates of over 98% for NH_4_^+^-N (Figure 4E), and 100% for both NO_2_^−^-N (Figure 5E) and NO_3_^−^-N (Figure 6E), at shaker speeds from 0 to 150 rpm. However, when the shaker speed increased to 200 rpm, the removal rates for all three nitrogen forms decreased. Specifically, the NH_4_^+^-N removal rate dropped to 92.48%, while the removal rates for NO_2_^−^-N and NO_3_^−^-N remained above 98%.

For HNAD bacteria, the heterotrophic nitrification and aerobic denitrification processes have different DO demands. Heterotrophic nitrification requires a relatively high DO concentration, whereas aerobic denitrification, particularly the reduction in NO_2_^−^ and subsequent steps, is favored under lower DO conditions [6]. The decline in nitrogen removal efficiency at 200 rpm can be attributed to two potential factors: (1) the high shaker speed may have caused physical shear stress and cell damage due to collisions [39], and (2) the correspondingly high DO level could inhibit the activity of denitrifying enzymes such as nitrite reductase (Nir), nitric oxide reductase (Nor), and nitrous oxide reductase (Nos) [6]. Although efficient nitrogen removal was also attained at lower shaker speeds, considering the requirement for higher biomass yield, 150 rpm was determined to be the optimal shaker speed for strain MJ20 cultivation.

#### 3.3.6. Salinity

Strain MJ20 was capable of growing within a salinity range of 0‰ to 50‰. When NH_4_^+^-N served as the sole nitrogen source (Figure 4F), its removal rate remained above 99% at salinities from 0‰ to 40‰, although the growth yield decreased with increasing salinity, reaching only 0.72 at 40‰. When salinity exceeded 40‰, both the removal rate and growth yield declined sharply. With NO_2_^−^-N (Figure 5F) or NO_3_^−^-N (Figure 6F) as the sole nitrogen source, the removal rates remained above 96% and 100%, respectively, within 0‰ to 35‰ salinity, but decreased markedly once salinity surpassed 35‰; the growth yield also exhibited a consistent declining trend with increasing salinity. In summary, for efficient nitrogen removal, it is recommended to maintain salinity between 0‰ and 40‰ for NH_4_^+^-N removal, and between 0‰ and 35‰ for NO_2_^−^-N and NO_3_^−^-N removal.

Excessively high salinity elevates osmotic pressure, which can adversely affect the activity of metabolic enzymes and consequently impair both bacterial growth and nitrogen removal performance [40]. Moreover, even for the same strain, the optimal salinity range often differs between the removal of NH_4_^+^-N and that of NO_2_^−^-N/NO_3_^−^-N [6], a pattern clearly observed with strain MJ20. The strain demonstrated efficient nitrogen removal and robust growth within 0–35‰ salinity, indicating its potential suitability for aquaculture applications across freshwater, brackish water, and seawater environments. Furthermore, this broad salt tolerance suggests that strain MJ20 could maintain stable performance in practical scenarios where salinity frequently fluctuates, such as in estuarine aquaculture, during rainy seasons when seawater salinity drops, or in intensive aquaculture systems where salinity is actively managed. This adaptability may reduce the risk of system instability due to environmental variations, making it a promising candidate for bioremediation in diverse and dynamic aquaculture settings.

#### 3.3.7. Nitrogen Concentration

Strain MJ20 exhibited significant differences in its growth and nitrogen removal characteristics when supplied with different nitrogen sources and concentrations. When NH_4_^+^-N served as the sole nitrogen source (Figure 4G), both the biomass and NH_4_^+^-N removal rate gradually decreased as its concentration increased from 100 mg/L to 1000 mg/L. At concentrations of 100–200 mg/L, NH_4_^+^-N removal exceeded 99% and biomass reached above 2.1, whereas at 1000 mg/L, the removal rate was only 10.85% and biomass dropped to 0.13. When NO_2_^−^-N was supplied as the sole nitrogen source (Figure 5G), its removal rate reached 100% within 100–500 mg/L, but declined sharply to 16.78% at 1000 mg/L, accompanied by a decrease in biomass from 1.68 to 0.47. Similarly, with NO_3_^−^-N as the sole nitrogen source (Figure 6G), the removal efficiency was 100% at 100–500 mg/L and remained at 80% at 600 mg/L; however, it dropped sharply to 4.12% at 1000 mg/L, with biomass declining from 1.43 to 0.45. Notably, the removal capacity of MJ20 (500 mg/L for both NO_2_^−^-N and NO_3_^−^-N) was remarkably higher than the maximum removal capabilities of *Pseudomonas stutzeri* YZN-001 [41] (275.08 mg/L for NO_2_^−^-N and 171.40 mg/L for NO_3_^−^-N) and *Pseudomonas* sp. B6-2 [42] (284.6 mg/L for NO_2_^−^-N and 187.54 mg/L for NO_3_^−^-N). In summary, to achieve efficient nitrogen removal by strain MJ20, the recommended initial concentrations are below 200 mg/L for NH_4_^+^-N, below 500 mg/L for NO_2_^−^-N, and below 600 mg/L for NO_3_^−^-N.

Among the different nitrogen sources, NH_4_^+^-N exhibits the strongest toxicity. Molecular ammonia (NH_3_), due to its lipid solubility, can penetrate the cell membrane and inhibit enzymatic activity, thereby disrupting cellular metabolism [43]. NO_2_^−^-N is also relatively toxic, as it can suppress cell wall synthesis, alter membrane permeability, and facilitate the uptake of harmful substances [44]. In contrast, NO_3_^−^-N shows lower toxicity [45]. As a result, HNAD bacteria generally exhibit higher tolerance to NO_3_^−^-N, a pattern that is consistent with the tolerance trend observed for strain MJ20: NO_3_^−^-N > NO_2_^−^-N > NH_4_^+^-N. Furthermore, although strain MJ20 showed varying tolerance thresholds for the different nitrogen forms, it was capable of completely degrading each at 100 mg/L within 24 h. Since such high levels of toxic nitrogen are rarely encountered in typical aquaculture environments, this strain demonstrates strong potential to serve as a dominant microbial candidate in biofloc technology for effective aquaculture water quality management.

### 3.4. Nitrogen Removal Characteristics Under Different Nitrogen Sources

#### 3.4.1. NH_4_^+^-N as Sole Nitrogen Source

**Figure 7:**
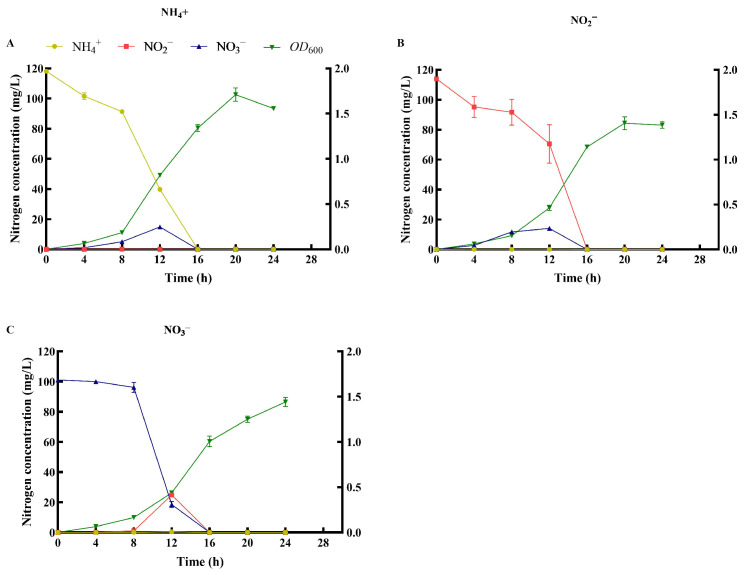
Nitrogen removal characteristics under different sole nitrogen sources. (**A**) NH_4_^+^; (**B**) NO_2_^−^; (**C**) NO_3_^−^.

Figure 7A demonstrates that when NH_4_^+^-N served as the sole nitrogen source, it was completely removed (100%) within 16 h. The maximum degradation rate occurred between 8 and 12 h, with an average rate of 12.89 ± 0.45 mg/L/h, which exceeds that reported for *Pseudomonas aeruginosa* SH3 (4.24 mg/L/h) [33] and *Pseudomonas aeruginosa* P-1 (9.29 mg/L/h) [46]. During this period, transient nitrate accumulation was observed, peaking at 14.81 mg/L at 12 h; the accumulated nitrate was subsequently completely degraded by 16 h. Nitrite accumulation was almost undetectable throughout the process. Strain MJ20 achieved a maximum OD_600_ of 1.71 among the seven nitrogen source combinations tested, reaching this density at 20 h before entering the stationary phase. Under the optimal conditions, the specific growth rate of the strain MJ20 was determined to be 0.139 h^−1^, with a corresponding doubling time of approximately 5.0 h during the exponential phase. The observed transient accumulation of nitrate is a common feature in the heterotrophic nitrification process of HNAD bacteria [14], confirming the occurrence of ammonia oxidation in this strain.

#### 3.4.2. NO_2_^−^-N as Sole Nitrogen Source

As depicted in Figure 7B, when NO_2_^−^-N served as the sole nitrogen source, it was also completely removed (100%) within 16 h. The maximum degradation rate occurred between 12 and 16 h, with an average rate of 17.64 ± 3.2 mg/L/h, exceeding that reported for *Halomonas venusta* SND-01 (5.95 mg/L/h) [47], *Pseudomonas aeruginosa* SH3 (2.47 mg/L/h) [33] and *Pseudomonas aeruginosa* P-1 (3.72 mg/L/h) [46]. During this process, transient nitrate accumulation was observed, peaking at 14.02 mg/L at 12 h, and the accumulated nitrate was subsequently completely degraded by 16 h. Ammonia nitrogen accumulation was almost undetectable throughout the process. The biomass of strain MJ20 reached a maximum OD_600_ of 1.41 at 20 h, which was lower than that observed in the NH_4_^+^-N treatment group. The accumulation of nitrate during the degradation of both NH_4_^+^-N and NO_2_^−^-N suggests the occurrence of heterotrophic nitrification in the nitrogen removal pathway of this strain. Specifically, the accumulation of nitrate when NO_2_^−^-N was the sole substrate strongly indicates that strain MJ20 possesses nitrite oxidation activity, capable of converting part of the nitrite to nitrate. Combined with the observation of nitrate accumulation during NH_4_^+^-N degradation, it is plausible that strain MJ20 harbors a relatively complete nitrification capacity, transforming NH_4_^+^ to NO_2_^−^ and further to NO_3_^−^.

#### 3.4.3. NO_3_^−^-N as Sole Nitrogen Source

When NO_3_^−^-N served as the sole nitrogen source, it was completely removed (100%) within 16 h (Figure 7C). The maximum degradation rate occurred between 8 and 12 h, with an average rate of 19.42 ± 1.15 mg/L/h, exceeding that reported for *Bacillus thuringiensisstrain* WXN-23 (5.62 mg/L/h) [14], *Pseudomonas fluorescens* 2P24 (4.29 mg/L/h) [48] and *Pseudomonas aeruginosa* P-1 (6.12 mg/L/h) [46]. During this process, transient nitrite accumulation was observed, peaking at 24.89 mg/L at 12 h, and the accumulated nitrite was subsequently completely degraded by 16 h. Ammonia accumulation was nearly undetectable throughout the process. The biomass of strain MJ20 reached a maximum OD_600_ of 1.44 at 24 h, which was comparable to the level attained in the NO_2_^−^-N treatment group. The transient accumulation of nitrite indicates that strain MJ20 is capable of reducing NO_3_^−^-N to NO_2_^−^-N under aerobic conditions, a key step in the aerobic denitrification pathway.

Collectively, the results from Figure 7A–C provide strong evidence that strain MJ20 harbors a complete HNAD metabolic network. Under the defined experimental context (glucose as sole carbon source, C/N = 15, pH 8, 30 °C, salinity 0‰, 150 rpm), the strain demonstrated its “all-in-one” metabolic capability by achieving 100% removal of 100 mg/L of each of the three common inorganic nitrogen forms (NH_4_^+^-N, NO_2_^−^-N, and NO_3_^−^-N). Specifically, it can oxidize NH_4_^+^-N/NO_2_^−^-N to NO_3_^−^-N (nitrification, Figure 7A,B) and reduce NO_3_^−^-N to NO_2_^−^-N and subsequently to gaseous nitrogen (denitrification, Figure 7C). This metabolic versatility enables the strain to independently and efficiently initiate nitrogen removal from any of the three common inorganic nitrogen forms and achieve complete nitrogen elimination. This “all-in-one” capability presents a significant advantage for treating aquaculture water or wastewater characterized by complex and variable nitrogen compositions.

#### 3.4.4. Nitrogen Removal Performance with Combined Nitrogen Sources

**Figure 8:**
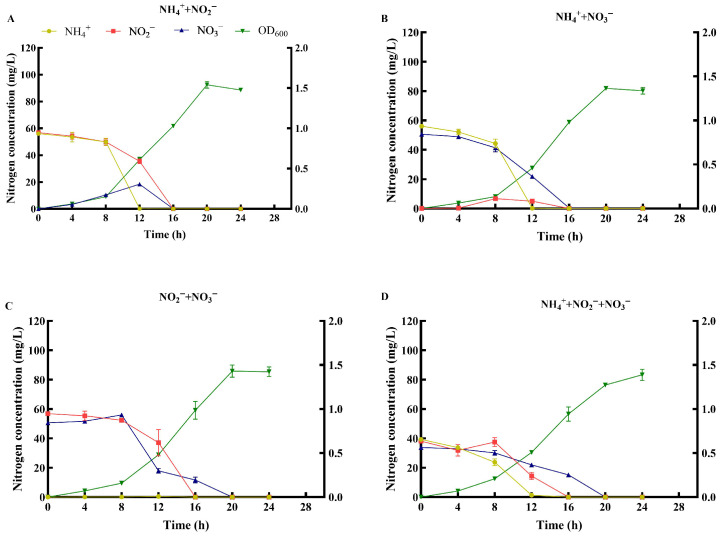
Nitrogen removal characteristics under different combined nitrogen sources. (**A**) NH_4_^+^ + NO_2_^−^; (**B**) NH_4_^+^ + NO_3_^−^; (**C**) NO_2_^−^ + NO_3_^−^; (**D**) NH_4_^+^ + NO_2_^−^ + NO_3_^−^.

Strain MJ20 demonstrated efficient removal of composite nitrogen sources. When NH_4_^+^-N and NO_2_^−^-N coexisted, the total nitrogen (112.96 mg/L) was completely removed within 16 h (Figure 8A). NH_4_^+^-N and NO_2_^−^-N were eliminated within 12 h and 16 h, respectively, with maximum average removal rates of 12.41 mg/L/h and 8.85 mg/L/h. Transient nitrate accumulation was observed but was cleared by 16 h. With NH_4_^+^-N and NO_3_^−^-N as nitrogen sources, the total nitrogen (106.67 mg/L) was completely removed within 16 h (Figure 8B). NH_4_^+^-N was removed within 12 h (maximum average rate: 11.01 mg/L/h, and NO_3_^−^-N was removed within 16 h (maximum average rate: 5.39 mg/(L·h)), accompanied by temporary nitrite accumulation. In the presence of both NO_2_^−^-N and NO_3_^−^-N, the total nitrogen (107.38 mg/L) was completely removed within 20 h (Figure 8C). NO_2_^−^-N and NO_3_^−^-N were eliminated within 16 h and 20 h, with maximum average rates of 9.26 mg/L/h and 9.51 mg/L/h, respectively. When all three nitrogen sources were supplied simultaneously, the total nitrogen (111.31 mg/L) was completely removed within 20 h (Figure 8D). NH_4_^+^-N, NO_2_^−^-N, and NO_3_^−^-N were sequentially removed at 12 h, 16 h, and 20 h, with peak average removal rates of 5.61 mg/L/h, 5.77 mg/L/h, and 3.80 mg/L/h, respectively. Under all co-substrate conditions, strain MJ20 grew well, with biomass peaking between OD_600_ 1.34 and 1.71 at 20–24 h.

The removal patterns with mixed nitrogen sources provide further insight into the metabolic strategy of strain MJ20. As depicted in single-substrate experiments, the strain possesses both nitrification (evidenced by NO_3_^−^-N accumulation from NH_4_^+^-N or NO_2_^−^-N) and denitrification capabilities (evidenced by NO_2_^−^-N accumulation from NO_3_^−^-N). When supplied with mixed sources, strain MJ20 consistently utilized NH_4_^+^-N prior to NO_2_^−^-N or NO_3_^−^-N, a preference also reported in other HNAD bacteria like *Ochrobactrum anthropi* HND19 [49] and *Acinetobacter* sp. JR1 [50]. This sequential removal suggests a regulatory mechanism that prioritizes the assimilation or oxidation of reduced nitrogen for growth. As depicted in Figure 8D, the rapid decrease in NH_4_^+^-N concentration (0–12 h) coincided with the exponential increase in biomass (OD_600_), indicating that the strain preferentially utilized NH_4_^+^-N to support cell proliferation. The utilization of NO_2_^−^-N or NO_3_^−^-N only became significant after NH_4_^+^-N depletion, likely to avoid the higher energy cost associated with nitrate assimilation during the active growth phase.

In the combination of NO_2_^−^-N and NO_3_^−^-N, a short-term increase in NO_3_^−^-N concentration was observed, likely due to the oxidation of NO_2_^−^-N via heterotrophic nitrification preceding denitrification. A transient rise in NO_2_^−^-N occurred in the three-source mix. This can be explained by electron competition within the respiratory chain: NO_3_^−^-N is a stronger competitor for electrons derived from quinones or cytochromes than NO_2_^−^-N, leading to its preferential reduction once a certain threshold is reached, which temporarily suppresses NO_2_^−^-N reduction [51]. Concurrently, the presence of NH_4_^+^-N may upregulate the transcription of the nitrate reductase gene *napA*, channeling nitrogen flux toward the ammonium assimilation pathway to optimize biomass synthesis [52].

Based on a comprehensive analysis of the removal behavior of strain MJ20 with both single and mixed nitrogen sources, its efficient metabolic logic for dealing with complex nitrogen pollution can be delineated. This strain exhibits a clear substrate utilization priority and a pathway coordination strategy: it preferentially assimilates or oxidizes ammonium nitrogen (NH_4_^+^-N) to support biomass synthesis and reduce system toxicity, followed by the synergy and competition between nitrification and denitrification pathways to achieve complete total nitrogen removal. This temporal sequence of “assimilation/oxidation first, reduction later”, coupled with the “electron competition regulation” mechanism (i.e., the competition among different nitrogen oxides as electron acceptors in the respiratory chain), forms the core physiological basis for its efficient treatment of complex nitrogen pollution. This strategy guarantees that the strain can optimize its growth while effectively and comprehensively accomplishing nitrogen removal in variable environments, underscoring its potential for practical application in aquaculture water or wastewater treatment scenarios.

#### 3.4.5. Maximum Average Degradation Rates Using Different Nitrogen Sources

**Figure 9:**
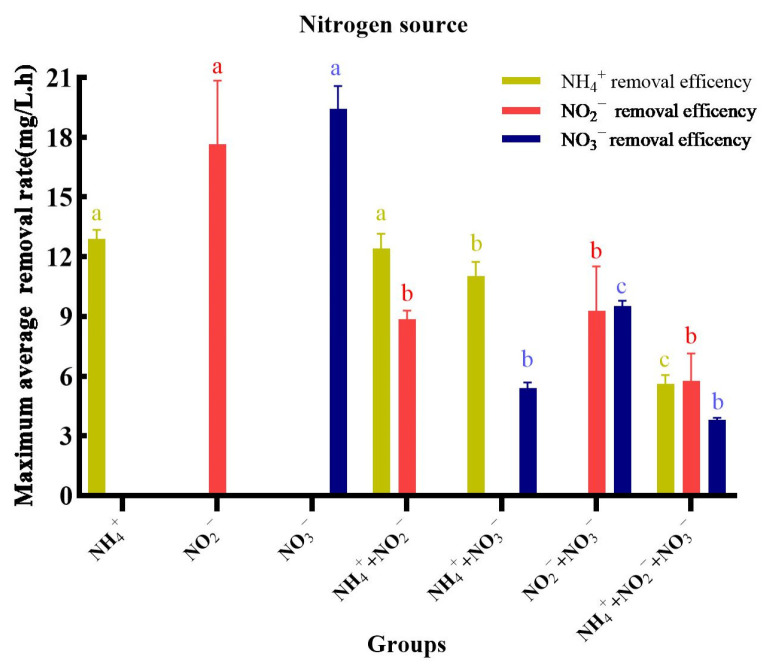
Maximum average degradation rates using different nitrogen sources. Treatments labeled with different letters are significantly different (*p* < 0.05).

The data in Figure 9 indicates that in the single nitrogen source experiments, NO_3_^−^-N exhibited the highest maximum average removal rate, while NH_4_^+^-N showed the lowest. However, the maximum removal rates for all nitrogen forms in mixed-substrate experiments were lower than when they served as the sole nitrogen source. In the two-source mixtures, NH_4_^+^-N demonstrated the highest maximum removal rate, exceeding that of the other nitrogen form present. In the combination of NO_2_^−^-N and NO_3_^−^-N, their maximum removal rates were comparable, and both were higher than their respective rates when mixed with NH_4_^+^-N. When all three nitrogen sources were mixed, NO_3_^−^-N had the lowest maximum removal rate, while the rates for NH_4_^+^-N and NO_2_^−^-N were similar.

This pattern can be explained by the differential energy demands of nitrogen assimilation. NH_4_^+^-N is a more direct and energetically favorable nitrogen source for cellular assimilation [53]. In contrast, NO_2_^−^-N or NO_3_^−^-N must undergo a series of energy-intensive reduction steps to ammonium before incorporation into biomolecules like amino acids [54]. Consequently, in mixed nitrogen environments, strain MJ20 preferentially and rapidly utilized NH_4_^+^-N to meet its growth demands, which explains its higher removal rate in such settings. The lower removal rates in mixtures, compared to single-substrate conditions, are likely due to metabolic competition for shared resources (e.g., reducing equivalents, carbon skeletons), and potential regulatory cross-talk between the parallel nitrification and denitrification pathways [14,55].

### 3.5. Nitrogen Balance Analysis

A nitrogen balance analysis of strain MJ20’s removal process across seven nitrogen source conditions is summarized in Table 2. When NH_4_^+^-N served as the sole nitrogen source, approximately 77.15% was assimilated into biomass, a rate higher than that observed with NO_2_^−^-N or NO_3_^−^-N as the sole source (58.14% and 62.23%, respectively). This finding aligns with reports for other bacteria, such as *Rhodococcus erythropolisstrain* Y10 [56]. The high assimilation rate (77.15% with NH_4_^+^-N) is superior to many other reported strains such as *Alphaproteobacteria* W30 (64.70%) [57] and *Acinetobacter johnsonii* EN-J1 (24.41%) [58], suggesting that MJ20 is particularly effective at converting toxic nitrogen into microbial protein, a desirable trait for single-cell protein production in aquaculture. In these single-source experiments, the remaining nitrogen—approximately 22.85%, 41.86%, and 37.77% for NH_4_^+^-N, NO_2_^−^-N, and NO_3_^−^-N, respectively—was presumed to be lost as gaseous products via denitrification. Total nitrogen removal reached 100% in all single-source groups, surpassing the values reported in many HNAD strains [57,58,59]. In the mixed nitrogen source groups, the NH_4_^+^-N and NO_2_^−^-N combination yielded the highest assimilation rate (~62.24%), followed by the NO_2_^−^-N and NO_3_^−^-N combination (~58.34%). Both rates exceeded those of the NH_4_^+^-N and NO_3_^−^-N group and the three-nitrogen mixture group. Total nitrogen removal remained at 100% across all mixed-source conditions.

Therefore, strain MJ20 primarily removed nitrogen via assimilation when supplied with single nitrogen sources or with the NH_4_^+^ + NO_2_^−^ and NO_2_^−^ + NO_3_^−^ combinations. However, in the NH_4_^+^ + NO_3_^−^ mixture and the three-nitrogen mixture, the denitrification pathway became dominant. This indicates that different nitrogen source combinations may shift the metabolic flux between assimilation and denitrification, a regulatory phenomenon also observed in bacteria like *Pseudomonas aeruginosa* P-1 [46].

### 3.6. Analysis of the Nitrogen and Carbon Metabolic Pathway

#### 3.6.1. Nitrogen Metabolic Pathway Analysis

The nitrogen metabolic pathway of MJ20 was investigated via complete genome sequencing. Based on the annotation results of the complete genome sequence, nitrogen assimilation and nitrogen denitrification are the primary nitrogen removal pathways for bacterial strain MJ20.

**Figure 10:**
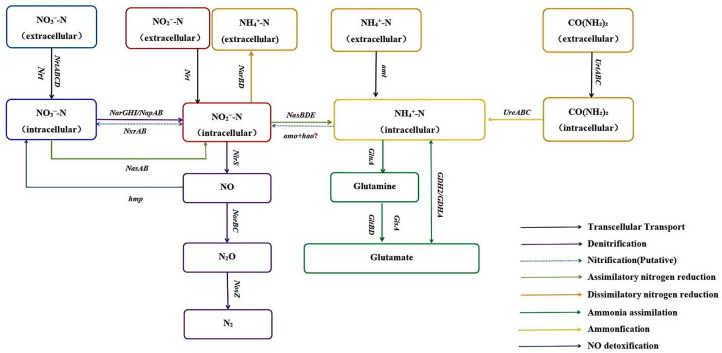
Predicted nitrogen metabolism map of strain MJ20.

Strain MJ20 possesses an efficient and diverse genetic foundation for nitrogen metabolism. The predicted nitrogen metabolic pathways are summarized in Figure 10. First, it harbors a complete urease system (*UrtABC* and *UreABC*) for urea transport and hydrolysis into ammonia. For ammonia assimilation, key genes for both the Glutamine Synthetase-Glutamate Synthase (GS-GOGAT) pathway (*glnA*, *gltBD*) and the Glutamate Dehydrogenase (GDH) pathway (gdh) are present, enabling efficient glutamate synthesis for protein production. Regarding nitrate utilization, a comprehensive network is evident: extracellular nitrate can be imported via transporters (e.g., nrt) and reduced to nitrite by the periplasmic nitrate reductase (encoded by *napAB*) under aerobic conditions or by the membrane-bound nitrate reductase (encoded by *narGHI*) under anaerobic conditions. The presence of both *napAB* and *narGHI* indicates that MJ20 possesses the genetic potential for nitrate reduction under both aerobic and anaerobic conditions. The resulting nitrite can be metabolized through three major branches: (1) dissimilatory nitrate reduction to ammonium (DNRA), via dissimilatory nitrite reductase (*nirBD*); (2) assimilatory nitrate reduction to ammonium, via assimilatory nitrite reductase (*nasBDE*); or (3) denitrification to N_2_, via the canonical *nirS*(cytochrome cd_1_-type nitrite reductase) → *norBC*(nitric oxide reductase) → *nosZ*(nitrous oxide reductase) pathway.

Second, the strain’s genetic repertoire suggests complementary and protective mechanisms for nitrogen metabolism. Genes encoding nitrite oxidoreductase (*nxrAB*) were annotated, whereas those for ammonia monooxygenase (*amoCBA*) and hydroxylamine oxidase (*hao*) were not detected, consistent with findings in related strains [33]. Notably, *nxrA* and *nxrB* in the KEGG database share functional unit annotations with *narG*/*narZ* and *narH*/*narY*, respectively, and no distinct *nxr* genes were annotated in the Nr database. Previous studies have indicated potential homology between *nxrAB* and *narGH* [60]. The presence of a complete *narGHIJ* gene cluster may therefore also confer genetic potential for nitrite oxidation to nitrate, aligning with the observed nitrate accumulation in our experiments (Section 3.4). The absence of annotated *amo* and *hao* genes may reflect their low sequence homology to those of canonical nitrifiers [61,62]. However, it also implies that the strain could utilize an alternative, non-classical pathway for ammonia oxidation that does not rely on the conventional *AMO*/*HAO* enzyme system.

Additionally, strain MJ20 encodes a nitric oxide dioxygenase (*hmp*), which detoxifies nitric oxide (NO) by converting it to nitrate in the presence of O_2_ [63]. This mechanism likely helps maintain cellular homeostasis under high-DO conditions where NO accumulation could inhibit nitric oxide reductase (Nor) activity, thereby supporting denitrification efficiency in oxygen-rich environments and overall system stability.

#### 3.6.2. Carbon Metabolic Pathway Analysis

**Figure 11:**
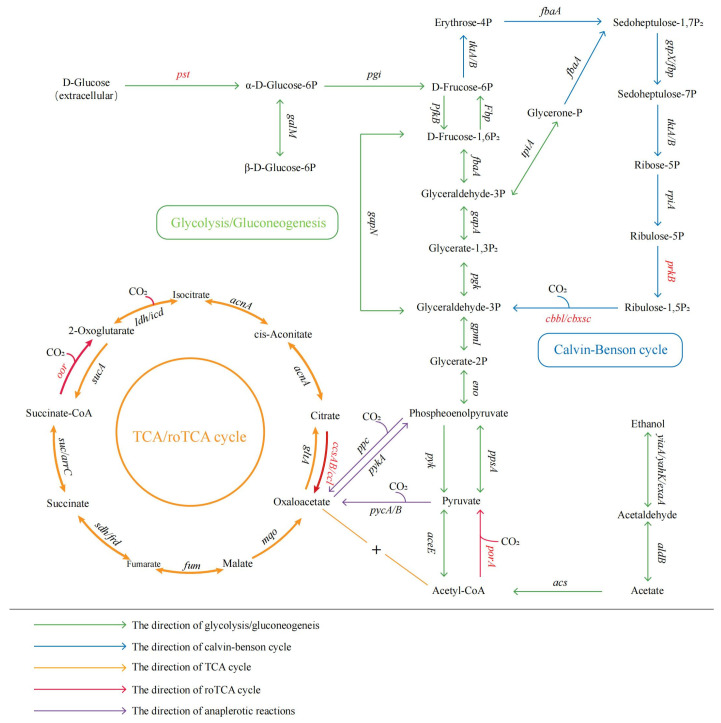
Predicted carbon metabolism map of strain MJ20. Note: genes that failed annotation are marked in red.

Comparative genomic analysis of strain MJ20 indicates a relatively complete glycolytic pathway, a full tricarboxylic acid (TCA) cycle, and partial pathways for the reverse TCA cycle and the Calvin–Benson–Bassham cycle (genes that failed annotation are marked in red in Figure 11).

The genome encodes a complete glycolytic pathway, enabling the catabolism of α-D-glucose, β-D-glucose, and their phosphorylated derivatives (e.g., α-D-glucose-1-phosphate, α-D-glucose-6-phosphate, β-D-glucose-6-phosphate) to pyruvate, which subsequently feeds into the TCA cycle. Notably, key genes for the phosphotransferase system (PTS), responsible for concomitant transport and phosphorylation of extracellular glucose, were not identified. This genomic prediction appears contradictory to experimental results, which confirmed efficient glucose utilization. This suggests the presence of alternative, PTS-independent transport and phosphorylation mechanisms. Furthermore, while genes for enzymes involved in sucrose, starch, and cellulose metabolism are present, corresponding genes for dedicated transmembrane transport systems are lacking. The observed, albeit moderate, utilization of sucrose and starch in experiments supports the hypothesis that strain MJ20 may secrete extracellular enzymes to hydrolyze these polymers into transportable monomers.

**Figure 12:**
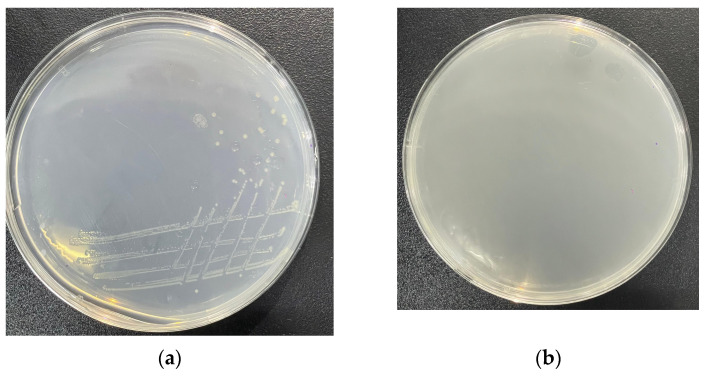
The growth of strain MJ20 on autotrophic plate with and without NaHCO_3_. Note: (**a**) Growth of strain MJ20 on autotrophic plate with NaHCO_3_; (**b**) Growth of strain MJ20 on autotrophic plate without NaHCO_3_.

Genes for all enzymes of the TCA cycle are present. Additionally, genes encoding transporters for acetate (*actP*), succinate (*dctMQ*), and citrate (*citMHS*) were identified. As these compounds are TCA cycle intermediates, the presence of dedicated transporters explains the strain’s rapid utilization of these organic acids. Although genes for segments of carbon fixation pathways (reverse TCA cycle, Calvin cycle) were found, key enzymes—including citrate lyase, α-ketoglutarate synthase, and pyruvate synthase for the reverse TCA cycle, and ribulose-1,5-bisphosphate carboxylase/oxygenase (Rubisco), phosphoribulokinase, and sedoheptulose-1,7-bisphosphatase for the Calvin cycle—were absent. This indicates limited potential for canonical autotrophic carbon fixation. However, strain MJ20 demonstrated growth on a HNAD basal medium containing no organic carbon but supplemented with 1.5 g/L sodium bicarbonate. Growth was confirmed on a solid inorganic autotrophic medium (HNAD basal medium with 100 mg/L NH_4_^+^-N, 1.5% agar, and sodium bicarbonate as the sole carbon source) (Figure 12a), while no growth occurred on a control medium lacking any added carbon source (Figure 12b) (incubation at 30 °C for 48 h). This confirms the strain’s ability to utilize bicarbonate for growth, though the specific enzymatic mechanisms require further investigation. But to verify the flux of inorganic carbon into central metabolites, future isotopic tracing (^13^C-HCO_3_^−^) experiments are necessary.

### 3.7. Analysis of the Flocculation Ability of Strain MJ20

**Figure 13:**
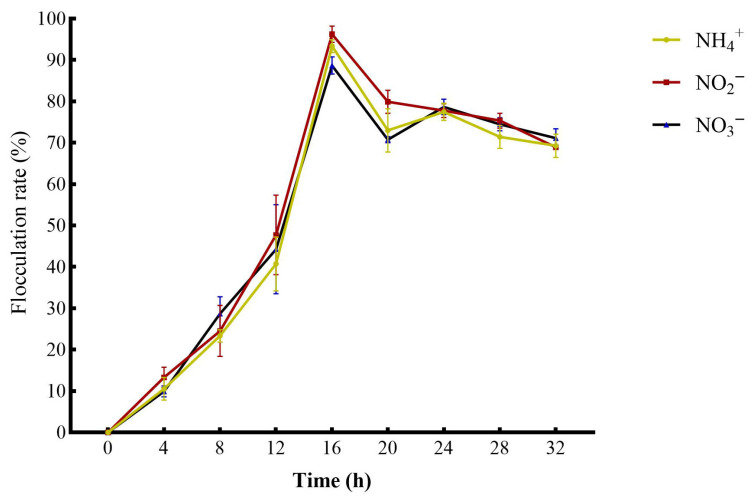
Dynamic changes in the flocculation rate of strain MJ20 in different types of nitrogen removal processes.

Strain MJ20 exhibited flocculating activity when cultured in media with ammonium sulfate, sodium nitrite, or potassium nitrate as the sole nitrogen source. The variation in its flocculation rate over time is shown in Figure 13. The flocculation ability of strain MJ20 increased continuously in media with different nitrogen sources over time, reaching a peak at 16 h. The highest flocculation rates were 96.25% in the sodium nitrite group, 93.37% in the ammonium sulfate group, and 88.69% in the potassium nitrate group. After 16 h, the flocculation rates in all groups began to decline, dropping to approximately 70% by the end of the cultivation period.

Flocculation-active strains secrete abundant EPS, including polysaccharides, proteins, and nucleic acids [64]. These EPS components efficiently aggregate dispersed organic debris, plankton, inorganic particles, and other microorganisms in water. This process provides a critical material foundation and structural framework for the development and functional maturation of bioflocs. Bioflocs can serve as an important source for isolating efficient flocculating bacteria [65]. The strain MJ20 isolated from bioflocs in this study exhibited strong flocculation ability. Its high flocculating activity implies enhanced capture and binding efficiency of suspended particles, which can promote the rapid enlargement of initial flocs through continuous adsorption and microbial proliferation within a short time. This characteristic underscores the significant application potential of MJ20 in enhancing biofloc formation and improving system stability.

### 3.8. Biosafety Analysis of Strain MJ20

#### 3.8.1. Hemolysis Assay

**Figure 14:**
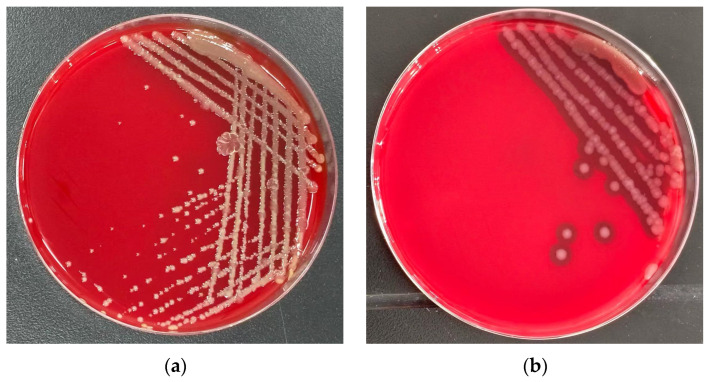
Hemolytic activity of strain MJ20. Note: (**a**) No hemolytic of strain MJ20; (**b**) Beta-hemolytic of *Vibrio parahaemolyticus*.

The results of the hemolytic activity assay are shown in Figure 14. No hemolytic zone was observed around strain MJ20 on the blood agar plate (Figure 14a), while the positive control, *Vibrio parahaemolyticus*, exhibited a typical β-hemolytic zone (Figure 14b). These results indicate that strain MJ20 is non-hemolytic.

#### 3.8.2. Antimicrobial Susceptibility Testing

In the results, S represents Sensitive (susceptible to treatment), M represents Intermediate (meaning the strain is inhibited by usually attainable concentrations of the antibiotic in specific body sites or when high dosage is used), and R represents Resistant. The antimicrobial susceptibility testing results (Table 3) showed that strain MJ20 was susceptible to most of the 15 antibiotics tested, exhibiting resistance only to penicillin, cefalexin, and cefradine. The observed resistance to penicillin, cephalexin, and cefradine (first generation cephalosporins), as well as the reduced susceptibility to ampicillin, erythromycin, chloramphenicol and co-trimoxazole, is consistent with the intrinsic resistance profile characteristic of the *Pseudomonas* genus. This phenotype is primarily attributed to the low permeability of the outer membrane (porins), the constitutive expression of chromosomal AmpC β-lactamases, and the activity of multidrug efflux pumps (e.g., MexAB-OprM) [66,67,68]. Furthermore, the resistance to co-trimoxazole is also consistent with the presence of chromosomally encoded antibiotic-inactivating enzymes documented in *Pseudomonas aeruginosa* [69], such asdihydropteroate synthase, which exhibits low affinity for sulfonamides, thereby bypassing the metabolic blockade intended by the drug.

Although the strain was resistant to several antibiotics mentioned above, the isolate still remained sensitive to carbenicillin, ceftriaxone, doxycycline, and other antibiotics, indicating that it does not possess a broad-spectrum multidrug resistance profile that would pose a severe untreatable risk. However, the potential for horizontal gene transfer of resistance determinants cannot be entirely ruled out without genomic analysis. Therefore, the application of this strain should be monitored, and further studies characterizing the genetic basis (chromosomal vs. plasmid-mediated) of this resistance are recommended in future work.

#### 3.8.3. Safety Assessment in an Aquatic Animal Model

The immersion challenge test employed in this study is a well-established method for evaluating the biosafety of microorganisms, as it mimics the natural route of infection through skin and gills [70]. In the immersion challenge test of zebrafish with strain MJ20, no mortality was observed in either the experimental or control groups over a 10-day observation period. Throughout the experiment, all zebrafish exhibited active feeding, normal growth, and no abnormal clinical signs attributable to the bacterial challenge. The absence of mortality in zebrafish challenged with strain MJ20 is consistent with the safety profiles of other *Pseudomonas* strains candidates [71,72,73]. These safety observations support the potential suitability of strain MJ20 as a probiotic candidate for aquaculture.

In summary, strain MJ20 demonstrated favorable comprehensive biosafety. The absence of hemolytic activity suggests a lack of exotoxin production, a primary virulence factor. This in vitro result was strongly supported by the in vivo zebrafish immersion challenge, which showed no mortality or clinical signs of disease. Moreover, the antibiotic susceptibility profile indicates that strain MJ20 lacks acquired resistance, thereby minimizing the risk of horizontal transfer of resistance genes. Collectively, these findings from antibiotic susceptibility testing, hemolysis assay, and animal challenge experiments indicate that strain MJ20 meets the rigorous safety criteria for a probiotic candidate in aquaculture.

### 3.9. Nitrogen Removal Performance in Simulated Mariculture Wastewater

**Figure 15:**
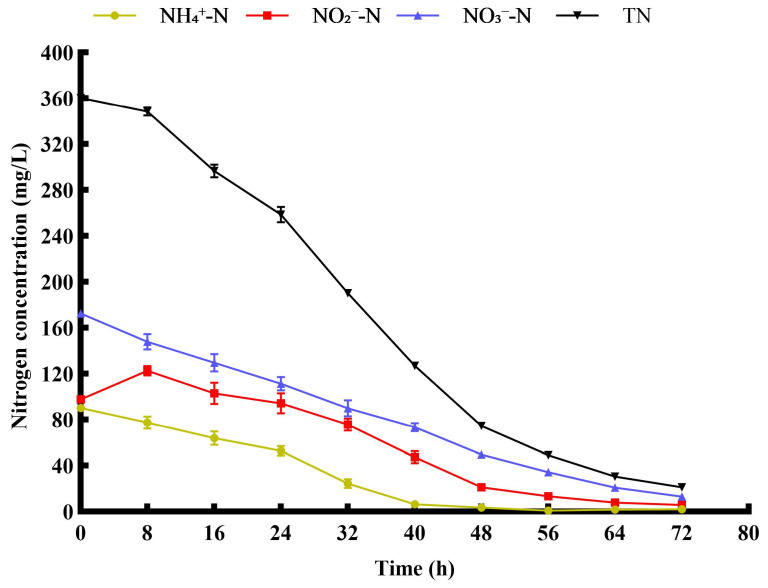
Nitrogen removal performance of MJ20 in simulated mariculture wastewater.

The nitrogen removal performance of strain MJ20 in simulated mariculture wastewater with a low C/N ratio (C/N = 5:1) is shown in Figure 15. The initial concentrations of NH_4_^+^-N, NO_2_^−^-N, NO_3_^−^-N, and TN in the wastewater were 90.27, 97.77, 172.47, and 360.5 mg/L, respectively. During the degradation process, NH_4_^+^-N was removed most rapidly, decreasing to its lowest level of 1.14 mg/L at 56 h, after which it remained relatively stable. This trend is consistent with the removal behavior of NH_4_^+^-N in the presence of other nitrogen sources described in Section 3.4. NO_2_^−^-N accumulated within the first 24 h of the reaction and then gradually decreased, reaching a final concentration of 5.96 mg/L. This transient accumulation may be attributed to the stronger electron competitiveness of NO_3_^−^ [51] during its reduction to NO_2_^−^, leading to a temporary increase in nitrite. NO_3_^−^-N and TN showed similar decreasing trends over 72 h, with their concentrations declining to 13.17 mg/L and 21.2 mg/L, respectively. At the end of the reaction, the removal rates of NH_4_^+^-N, NO_2_^−^-N, NO_3_^−^-N, and TN were 97.7%, 93.9%, 92.36%, and 94.12%, respectively. These results demonstrate that strain MJ20 exhibits efficient denitrification capability and holds practical application potential for treating marine aquaculture wastewater.

### 3.10. Potential Conditions for Establishing the Biofloc System for Wastewater Treatment

Based on the physiological and biochemical characteristics of strain MJ20 characterized in this study, we propose a potential strategy for establishing a biofloc system for aquaculture wastewater treatment. The integration of our data suggests the following optimal conditions for system operation.

#### 3.10.1. Optimization of Carbon Source and C/N Ratio

The addition of cost-effective organic carbon sources, such as glucose, is recommended to elevate the system’s C/N ratio to at least 10:1. This condition effectively promotes the proliferation of strain MJ20 and stimulates EPS secretion, thereby accelerating the rapid formation and growth of bioflocs. The resulting flocs efficiently adsorb and entrap functional strains (e.g., the HNAD bacterium identified in this study) alongside suspended and colloidal pollutants from the wastewater. This entrapment minimizes the washout of functional biomass in continuous-flow processes, maintaining a high concentration of active biomass within the system. Thus, a well-developed floc structure facilitates sludge settling, significantly reducing the concentration of suspended solids in the effluent and improving overall water quality.

#### 3.10.2. Control of Key Environmental Parameters

To ensure optimal metabolic activity and denitrification performance of strain MJ20, the system should be operated within the following parameters: temperature of 20–35 °C, pH of 6.0–9.0, dissolved oxygen greater than 6 mg/L, and salinity below 35‰. The influent ammonia nitrogen concentration is recommended to be maintained below 200 mg/L, while nitrite and nitrate should be kept below 500 mg/L. Under these conditions, the functional strain can both thrive and efficiently perform heterotrophic nitrification–aerobic denitrification, ensuring high and stable nitrogen removal efficiency.

#### 3.10.3. Floc Microzone-Mediated Denitrification

Upon biofloc formation, oxygen transfer becomes limited inside them, creating low-oxygen zones at their core. This natural structure allows different bacteria to work together in different parts of the floc. We suggest growing the aerobic HNAD bacteria from this study alongside traditional anaerobic denitrifiers. The aerobic HNAD bacteria will live mainly on the floc surface, where they convert ammonia to nitrite and nitrate. The anaerobic bacteria can then thrive in the inner, low-oxygen zones, where they convert nitrate into nitrogen gas. This cooperative setup makes the overall nitrogen removal process more complete and efficient.

## 4. Conclusions

This study presents *Stutzerimonas stutzeri* MJ20, a novel HNAD strain with robust nitrogen removal efficiency and strong flocculation ability. Its capacity to simultaneously remove nitrogen with low-cost carbon sources (glucose, sucrose) and aggregate biomass offers a streamlined, eco-friendly strategy for biofloc culture and treating low C/N aquaculture wastewater, representing its significant potential for application in BFT and wastewater treatment. However, the current evaluation was limited to controlled laboratory conditions using simulated wastewater. The strain’s performance and ecological impact within complex, real-world aquaculture systems remain uncertain. Future research should validate MJ20 in pilot-scale applications and investigate the regulatory mechanisms governing its dual metabolic pathways and flocculation behavior to facilitate field implementation.

## Figures and Tables

**Table 1:** Water quality parameters of the synthetic mariculture wastewater.

Items	Concentration
NH_4_Cl	343.9 mg/L
NaNO_2_	482.8 mg/L
KNO_3_	1248.5 mg/L
KH_2_PO_4_	316.8 mg/L
Na_2_CO_3_	130 mg/L
salinity	25‰
trace-element liquor	5 mL/L
pH	8.0

**Table 2:** Nitrogen balance of strain MJ20 during the nitrogen removal process.

Nitrogen Source	DTN_i_ (mg/L)	N_Bio0_ (mg/L)	DTN_f_ (mg/L)	N_Bio1_ (mg/L)	N_g_ (mg/L)	Nitrogen Assimilation Rate (%)
NH_4_^+^-N	118.02	2.79 ± 0.24	0	93.85 ± 1.79 ^a^	26.96 ± 1.79 ^e^	77.15 ± 1.52 ^a^
NO_2_^−^-N	113.91	2.79 ± 0.24	0	69.02 ± 1.20 ^b^	47.69 ± 1.22 ^c^	58.14 ± 1.05 ^ab^
NO_3_^−^-N	101.17	2.79 ± 0.24	0	65.75 ± 0.84 ^b^	38.21 ± 0.84 ^d^	62.23 ± 0.83 ^ab^
NH_4_^+^-N + NO_2_^−^-N	112.96	2.79 ± 0.24	0	73.55 ± 1.44 ^b^	42.20 ± 1.46 ^d^	62.64 ± 1.30 ^abc^
NH_4_^+^-N + NO_3_^−^-N	106.67	2.79 ± 0.24	0	52.86 ± 1.96 ^c^	56.60 ± 1.92 ^b^	46.94 ± 1.84 ^bc^
NO_2_^−^-N + NO_3_^−^-N	107.38	2.79 ± 0.24	0	65.43 ± 0.49 ^b^	44.74 ± 0.49 ^c^	58.34 ± 0.46 ^cd^
NH_4_^+^-N + NO_2_^−^-N + NO_3_^−^-N	111.31	2.79 ± 0.24	0	53.10 ± 3.81 ^c^	60.67 ± 3.82 ^a^	45.19 ± 3.41 ^d^

Different superscript letters in the same row represent significant differences (*p* < 0.05).

**Table 3:** Antibiotic sensitivity test results of strain MJ20.

Antimicrobial Agents	Concentration per Disc (μg per Tablet)	Diameter (mm)	Results
Penicillin	10	7.08 ± 0.57	R
Ampicillin	10	16.73 ± 1.76	M
Carbenicillin	100	22.16 ± 0.78	S
Cefalexin	30	6.91 ± 0.07	R
Cefradine	30	7.31 ± 0.32	R
Ceftriaxone	30	30.03 ± 2.15	S
Cefoperazone	75	27.06 ± 2.45	S
Ceftazidime	30	27.53 ± 0.90	S
Gentamicin	10	24.77 ± 0.92	S
Erythromycin	15	20.83 ± 1.40	M
Chloramphenicol	30	13.11 ± 0.35	M
Minocycline	30	19.68 ± 0.96	S
Doxycycline	30	22.74 ± 0.89	S
Polymyxin B	30	19.17 ± 0.94	S
Amikacin	30	26.57 ± 0.22	S
Enrofloxacin	10	30.18 ± 1.44	S
Norfloxacin	10	40.16 ± 2.05	S
Co-trimoxazole	23.75	19.18 ± 0.59	M

S represents Sensitive, M represents Intermediate, and R represents Resistant.

## Data Availability

The data presented in this study are available on request from the corresponding authors due to commercial restrictions.
